# Cancer-associated fibroblast promotes tamoxifen resistance in estrogen receptor positive breast cancer via exosomal LncRNA PRKCQ-AS1/miR-200a-3p/MKP1 axis-mediated apoptosis suppression

**DOI:** 10.1186/s13046-025-03529-x

**Published:** 2025-09-30

**Authors:** Chenghui Wu, Xi Sun, Yujie Lu, Haoyu Wang, Zhengyuan Yu, Zheng Wang, Renhong Huang, Yihua Jin, Xing Chen, Haixia Xie, Yu Zong, Kunwei Shen, Min Jiang, Yujie Tang, Xiaosong Chen

**Affiliations:** 1 Department of General Surgery, Comprehensive Breast Health Center, Ruijin Hospital, Shanghai Jiao Tong University School of Medicine, 197 Ruijin Er Road, Shanghai, 200025 China; 2 Shanghai Key Laboratory of Reproductive Medicine, Department of Histoembryology, Genetics and Developmental Biology, Shanghai Jiao Tong University School of Medicine, Shanghai, China; 3 Institute of Aging & Tissue Regeneration, Ren-Ji Hospital, Shanghai Jiao Tong University School of Medicine, Shanghai, 200127 China; 4 Department of Internal Medicine, Wayne State University, Detroit, MI USA; 5 Department of Oncology, The First Affiliated Hospital of Soochow University, No.899 Pinghai Road, Gusu District, Suzhou, 215006 Jiangsu China

**Keywords:** Cancer-associated fibroblast, Exosomes, Breast cancer, Tamoxifen resistance, LncRNA PRKCQ-AS1, MKP1

## Abstract

**Background:**

Tamoxifen resistance remains a significant challenge in the endocrine therapy of estrogen receptor-positive (ER+) breast cancer. Cancer-associated fibroblasts (CAFs) facilitate therapeutic resistance through exosome secretion, yet the role of CAF-derived long non-coding RNAs (lncRNAs) in tamoxifen resistance remains underexplored. This study investigates the mechanism by which CAF-secreted exosomal lncRNA modulates tamoxifen resistance in ER + breast cancer.

**Methods:**

CAFs from ER + breast cancer patients and normal fibroblasts (NFs) from healthy control were isolated and cultured ex vivo. Exosomes were extracted from the supernatant of cultured cells using ultracentrifugation. RNA sequencing identified PRKCQ-AS1 as a candidate lncRNA, validated in tamoxifen-sensitive and resistant tumors. Functional assays (qPCR, RNA-seq, dual-luciferase reporter, and in vivo xenografts) elucidated the role of PRKCQ-AS1/miR-200a-3p/MKP1 axis in tamoxifen resistance. Prognostic correlations were assessed in clinical cohorts (*n* = 471) and TCGA/GEO datasets.

**Results:**

LncRNA PRKCQ-AS1 was markedly upregulated in tamoxifen-resistant tumor samples and was associated with poor prognosis in tamoxifen-treated ER + breast cancer patients but not in those treated with aromatase inhibitors. Exosomal PRKCQ-AS1 derived from CAFs transferred to ER + breast cancer cells, suppressing tamoxifen-induced apoptosis via MKP1 upregulation. Mechanistically, PRKCQ-AS1 acted as a molecular sponge for miR-200a-3p, relieving miR-200a-3p-mediated repression of MKP1. Elevated MKP1 inactivated the MAPK/JNK pathway, attenuating apoptosis. In vivo, PRKCQ-AS1 overexpression accelerated ER + breast tumor growth despite tamoxifen treatment. TGF-β-driven CAF transformation upregulated PAX5, a transcription factor enhancing PRKCQ-AS1 expression.

**Conclusions:**

This study identifies CAF-derived exosomal LncRNA PRKCQ-AS1 as a key mediator of tamoxifen resistance via the miR-200a-3p/MKP1 axis. Targeting this pathway may offer novel therapeutic strategies to overcome endocrine resistance in ER + breast cancer.

**Supplementary Information:**

The online version contains supplementary material available at 10.1186/s13046-025-03529-x.

## Background

Breast cancer is one of the most prevalent malignancies among women worldwide, with estrogen receptor-positive (ER+) breast cancer comprising approximately 70% of cases [1]. Tamoxifen, a selective estrogen receptor modulator (SERM), functions by competitively binding to estrogen receptors, thereby inhibiting ER-driven tumor proliferation [2]. As a cornerstone of endocrine therapy for decades, tamoxifen has significantly improved clinical outcomes for patients with ER + disease [3]. However, the development of resistance remains a major challenge, often leading to disease recurrence and metastasis [4]. Several mechanisms contributing to tamoxifen resistance have been identified, including mutations or dysregulation of the estrogen receptor 1 (ESR1) gene, activation of the phosphatidylinositol 3-kinase-RAC-serine/threonine-protein kinase (PI3K-AKT) signaling pathway, and upregulation of activator protein-1 (AP-1) [5, 6]. Nevertheless, the precise molecular mechanisms underlying tamoxifen resistance remain incompletely understood, underscoring the need for further investigation.

Cancer-associated fibroblasts (CAFs), as key components of the breast cancer tumor microenvironment (TME), play a critical role in tumor progression, metastasis and therapeutic resistance. The intercellular crosstalk between cancer cells and CAFs is mediated by multiple factors, including cytokines, growth factors and chemokines [7]. One significant way in which CAFs deliver bioactive cargos to tumor cells is through exosomes [8, 9]. Exosomes are small extracellular vesicles, approximately 30–120 nm in diameter, that encapsulate diverse molecular cargo, including DNA, microRNA (miRNA), long non-coding RNA (lncRNA), and bioactive molecules, contributing to their functional heterogeneity [10]. By facilitating intercellular communication between stromal and tumor cells, exosomes significantly impact cancer progression and therapeutic response [11]. Notably, CAF-derived exosomal miR-22 and miR-20 have been shown to confer resistance to tamoxifen and abemaciclib in ER + breast cancer cells, respectively [12, 13]. However, additional components within CAF-derived exosomes that mediate tamoxifen resistance remain to be fully elucidated.

Among the molecular cargo within exosomes, non-coding RNAs play a pivotal role in epigenetic regulation, such as acting as a competitive endogenous RNA (ceRNA) or a molecular sponge for miRNAs, which is implicated in various oncogenic processes [14]. Emerging evidence suggests that non-coding RNAs contribute to tumorigenesis, progression, and drug resistance [15]. For instance, circKIF4A has been identified to promote brain metastasis in triple-negative breast cancer while circLIFR-007 appears to suppress liver metastasis in breast cancer [16, 17]. Previously, we reported that lncRNA ARA promoted adriamycin resistance in breast cancer [18]. However, the relationship between lncRNAs in CAF-derived exosomes and tamoxifen resistance in ER + breast cancer remains largely unexplored, potentially uncovering novel targets for overcoming resistance.

In this study, we aimed to elucidate the role of CAF-derived exosomes in mediating tamoxifen resistance in ER + breast cancer cells. Furthermore, we aimed to investigate mechanisms by which lncRNA contributes to tamoxifen resistance and to identify novel therapeutic targets within CAF-derived exosomal lncRNAs to overcome tamoxifen resistance in ER + breast cancer.

## Materials and methods

### Patient samples and clinical assessments

Tumor tissues from 471 ER + breast cancer patients were collected through surgery at the Comprehensive Breast Health Center, Ruijin Hospital, Shanghai Jiao Tong University School of Medicine, between July 2010 and July 2021 and then preserved by paraffin embedding. Among these, 68 tumor specimens from patients before adjuvant tamoxifen treatment were matched by clinicopathological features of patients, including tumor size, molecular subtype, histology, histological grade and Ki-67 index. Four paired frozen tamoxifen-sensitive and tamoxifen-resistant breast cancer samples were tested simultaneously to identify upregulated lncRNAs associated with tamoxifen resistance using bulk RNA sequencing (RNA-seq) (Fig. S1). The remaining 30 paired tumor samples were used to validate the identified upregulated lncRNAs via quantitative polymerase chain reaction (qPCR) assays. Besides, 403 ER + breast cancer samples were made into tissue microarray to assess the expression levels of PRKCQ-AS1 and MKP1 as Ruijin cohort. Additionally, survival outcomes and clinicopathologic characteristics of these ER + breast cancer patients were obtained for further analysis. Patients of these 403 tumor specimens received either tamoxifen or aromatase inhibitors treatment after surgery. Tamoxifen resistance is defined as patients who experience tumor recurrence or metastasis with tamoxifen adjuvant therapy. The study was approved by the Ethics Committee of Ruijin Hospital, Shanghai Jiao Tong University School of Medicine.

### Isolation and culture of CAFs and normal fibroblasts (NFs)

CAFs were isolated from seven human ER + breast cancer tissues before adjuvant tamoxifen treatment and NFs were isolated from the breast glandular tissue of three patients who underwent reduction mammaplasty. The culture process followed previously reported methods [19]. Briefly, breast cancer and normal breast tissues were mechanically cut into small pieces and digested with type IV collagenase (Gibco, #17104019) and hyaluronidase (Sigma, #HX0514). Cells were then suspended in DMEM/F-12 medium (Gibco, #11330-032) with 10% FBS and 1% PS. After subculturing for three generations, primary CAFs and NFs were purified using immunomagnetic bead cell sorting with anti-human fibroblast magnetic beads. CAFs and NFs were cultured in DMEM medium with 10% fetal bovine serum (FBS, Sigma, #F2442) and 1% penicillin-streptomycin (PS, BasalMedia, #S110JV), and cells from passages 4 to 10 were used for experiments.

### Cell culture and reagents

Human breast cancer cell lines (MCF7 and T47D) and HEK293T cells were obtained from the Shanghai Cell Bank, Chinese Academy of Sciences. All cells were cultured in DMEM high-glucose medium (BasalMedia, L110KJ) supplemented with 10% FBS and 1% PS solution. All cells were maintained at 37 °C in a humidified incubator with 5% CO2.

### Plasmid and transfection

The full-length human lncRNA PRKCQ-AS1 was cloned into pcDH-CMV vectors to create the pcDH-CMV-PRKCQ-AS1 plasmid. Short hairpin RNA (shRNA) plasmids targeting MKP1 were constructed by inserting target oligonucleotides into pLKO.1. Lentivirus was packaged by co-transfecting the plasmids with packaging vectors (pMD2.G and psPAX2) into HEK293T cells. The lentivirus was used to infect target cells to create stable transfecting cell lines. Primers for plasmid construction are listed in Table S1. PAX5 small interfering RNA (si PAX5#1, si PAX5#2), miR-200a-3p mimics and miR‐200a- inhibitor (sequences are listed in Supplementary Table S2) were designed and purchased from Servicebio (Wuhan, China). For each transfection well in 6-well plates, 200pmole of si PAX5#1, si PAX5#2, and the control group; 400pmole of miR-200a-3p mimics, miR-200a-3p inhibitor, and the control group were added to 500ul of serum-free medium and gently mixed. 5ul of Lipofectamine 3000 (Thermo Fisher Scientific, #L3000075) was added to the mixture above and incubated at room temperature for 20 min. The mixture was added to the 6-well plates containing cells with 1.5mL of serum-free medium and gently shaken to mix. The cells were cultured at 37 °C for 6 h and then the medium was replaced with complete medium. Cells were detected for transfection efficiency in 48 h after transfection.

### RNA extraction and quantitative polymerase chain reaction (qPCR) analysis

Total RNA from fresh tissue samples and cultured cells was extracted using Trizol reagent (Thermo Fisher Scientific, #AM9738). Total RNA from paraffin-embedded tissue was extracted using the PureLink™ FFPE RNA kit (Thermo Fisher Scientific, #K1560-02). Messenger RNAs (mRNAs) and lncRNAs were reverse transcribed to complementary DNA (cDNA) with the High-Capacity RNA-to-cDNA kit (Thermo Fisher Scientific, #4387406). The reverse transcription of miRNAs was performed using the PrimeScript RT Reagent Kit (Takara, #RR037a) with specific stem-loop primers. RNA expression was assessed in triplicate using real-time quantitative PCR on an Applied Biosystems QuantStudio™ 5 Real-Time PCR System (Thermo Fisher Scientific, #A34322) with SYBR Green Master buffer (ROX) (Thermo Fisher Scientific, #A25742). qPCR method for analyzing exosomal RNA, U6 was used as the internal control. For analyzing RNA in cells and tissues, expression levels of mRNAs and lncRNAs were normalized to GAPDH, and U6 was used as an internal control for miRNA expression. Primers for amplifying mRNAs, lncRNAs, and miRNAs are listed in Table S3.

### Western blotting (WB)

Total proteins were extracted from cell lysates using radioimmunoprecipitation assay (RIPA) buffer (Thermo Fisher Scientific, #89900) supplemented with 1% phenylmethyl sulfonyl fluoride (PMSF) (Thermo Fisher Scientific, #36978). Protein concentrations were quantified using the Pierce BCA kit (Thermo Fisher Scientific, #23225). Denatured proteins (20 µg/lane) were subjected to 10% SDS polyacrylamide gels and transferred onto polyvinylidene difluoride (PVDF) membranes (Thermo Fisher Scientific, #88518). Membranes were blocked with 5% fat-free milk (BD Biosciences, #232100) solution for 1 h at room temperature, then incubated with primary antibodies overnight at 4 °C. Membranes were then incubated with goat anti-rabbit secondary antibody (1:3000, Cell Signaling Technology, #98164) or goat anti-mouse secondary antibody (1:3000, Cell Signaling Technology, #91196). Protein detection was performed using enhanced chemiluminescence (ECL) reagents (Millipore, WBKLS0500). The primary antibodies used for WB are listed in Table S4.

### RNA sequencing (RNAseq)

RNA was extracted using the Universal RNA Extraction CZ Kit (RNC643, ONREW). RNA concentration was measured using Qubit 4.0 (Invitrogen), and quality was assessed by electrophoresis on a denaturing agarose gel. RNA libraries were prepared using the VVAHTS^®^ Universal V8 RNA-seq Library Prep Kit for Illumina (NR605-0, Vazyme) and sequenced on the Illumina NovaSeq 6000 platform. Raw sequencing data were processed using Skewer v0.2.2 (https://sourceforge.net/projects/skewer/files/?source=navbar) and quality checked with FastQC v0.11.2 (http://www.bioinformatics.babraham.ac.uk/projects/fastqc/). The read length was 2 × 150 bp. Clean reads were aligned to the human genome (GRCh38) using STAR (https://github.com/alexdobin/STAR). Gene expression data were generated with StringTie (v1.3.1c), and differential gene expression was analyzed using DESeq2 (v1.16.1) (https://bioconductor.org/packages/release/bioc/html/DESeq2.html). Differentially expressed genes (DEGs) were identified with thresholds of *P* < 0.05 and an absolute fold change ≥ 2. Gene set enrichment analysis (GSEA) was performed using R.

### Exosomes extraction and transmission electron microscope

As previously described [19], by seeding the same number of CAFs and NFs, same volume supernatants from NFs and CAFs cultures were collected after 24 h in serum-free medium. Supernatants were centrifuged at 2,000 × g for 20 min and 10,000 × g for 30 min at 4 °C. The resulting supernatant was transferred to ultracentrifugation tubes (Beckman Coulter, #355618) and centrifuged at 100,000 × g for 2 h at 4 °C using an ultracentrifuge (Beckman Coulter-Optima XPN-100). The exosome pellets were resuspended in PBS for further analysis.

### Immunofluorescence microscopy

CAFs and NFs were seeded onto glass coverslips and cultured for 48 h. Cells were fixed with 4% paraformaldehyde, blocked with 1% bovine serum albumin (BSA) for 1 h, and incubated overnight at 4 °C overnight with primary antibodies (listed in Table S4). The next day, cells were incubated for 1 h at room temperature with either anti-rabbit (1:1000, Cell Signaling Technology, #98164) or anti-mouse (1:1000, Cell Signaling Technology, #91196) secondary antibodies. Nuclei were counterstained with DAPI (Thermo Fisher Scientific, #62248). Stained samples were examined using confocal microscopy.

### Immunohistochemistry (IHC)

Formalin-fixed, paraffin-embedded (FFPE) tumor sections were rehydrated, and blocked with rabbit serum. The slides were incubated with primary antibodies at 4 °C overnight, followed by incubation with anti-rabbit secondary antibody (1:1000, Cell Signaling Technology, #98164) at 37 °C for 1 h. Primary antibodies are listed in Table S4. Staining was visualized using the Metal Enhanced DAB Substrate Kit (Dako, K5007) followed by hematoxylin counterstaining (Beyotime, C0105). Images were captured using an optical microscope.

### In situ hybridization (ISH)

FFPE tumor specimens were sectioned and subjected to ISH to detect PRKCQ-AS1 expression using a specific digoxigenin (DIG) labeled PRKCQ-AS1 probe. Images were captured using an optical microscope. The sequences for the DIG-labeled PRKCQ-AS1 probe are listed in Table S5.

The definition criteria for high and low PRKCQ-AS1 and MKP1 expression levels were determined based on the ISH (PRKCQ-AS1) and IHC (MKP1) pathological score in Ruijin cohort. IHC and ISH evaluations were conducted independently by at least two experienced pathologists from the Department of Pathology, Ruijin Hospital, Shanghai Jiao Tong University School of Medicine. IHC staining intensity was graded as: 0 (no staining), 1 (weak staining), 2 (moderate staining), and 3 (strong staining). The proportion of positively stained tumor cells was also assessed. The staining index (SI) was calculated by multiplying the staining intensity score by the proportion of positive tumor cells, yielding a final score ranging from 0 to 3. And scoring method of ISH is consistent with that of IHC. We used receiver operating characteristic (ROC) curve in R software to analyze the optimal cut-off value of PRKCQ-AS1 (0.65) and MKP1 (1.22). Besides, the criteria for GEO cohort was the optimal cut-off value of PRKCQ-AS1 (-2.34) and MKP1 (-1.86) using ROC curve. Breast cancer samples with PRKCQ-AS1 or MKP1 score higher than cut-off value were regarded as high levels, and vice versa as low levels.

### Cell-viability assays and colony-formation assays

Cell viability was assessed using the CellTiter-Glo^®^ luminescent cell viability assay (Promega, #G7573) according to the manufacturer’s protocol. Briefly, 3,000 cells per well were seeded into 96-well plates containing 100µL of culture media and evaluated after 72 h. For colony formation assays, 1,000 cells per well were seeded into 6-well plates, with the culture medium replenished every 4 days. Colonies were visible after 7–14 days, which were washed twice with PBS, fixed with 10% neutral formaldehyde solution, and stained with 0.5% crystal violet (Sigma, #C6158-100G) in 25% methanol.

### Flow cytometry

Apoptosis was assessed using the FITC Annexin V Apoptosis Detection Kit (MultiSciences, AT-101) according to the manufacturer’s instructions. Fluorescence-activated cell sorting (FACS) analysis was performed using a CytoFLEX FACS instrument (Beckman Coulter) and data were analyzed using FlowJo software.

### Dual-luciferase reporter assay

Dual-luciferase reporter assay was performed as previously described [20]. Wild-type and mutated sequences of PRKCQ-AS1 and MKP1 were cloned into the PmirGLO Vector to generate pmirGLO-PRKCQ-AS1-WT, pmirGLO-PRKCQ-AS1-MUT, pmirGLO-MKP1-WT, and pmirGLO-MKP1-MUT vectors. Negative control vectors (pmirGLO-PRKCQ-AS1-NC and pmirGLO-MKP1-NC) were used as references. HEK293T cells were co-transfected with these constructs and either miR-200a mimics or miRNA-NC using Lipofectamine™ 2000 (Thermo Fisher Scientific, #11668500). For the PRKCQ-AS1 promoter luciferase assay, HEK293T cells were co-transfected with the plasmids containing the putative binding sites of PAX5 to PRKCQ-AS1 promoter. Relative luciferase activity was measured 48 h post-transfection using the Dual-Luciferase Reporter Assay System (Promega, #E1910) according to the manufacturer’s instructions.

### In vivo assays

All animal procedures were approved by the Institutional Animal Care and Use Committee of Shanghai Jiao Tong University School of Medicine. Estrogen pellets (0.72 mg, 60-day slow release, Innovative Research of America) were implanted subcutaneously into the neck of female, 4-week-old NCG (NOD/ShiLtJGpt-Prkdc em26Cd52 Il2rg em26Cd22 /Gpt) mice (GemPharmatech Co., Ltd, Jiangsu, China). After one week, 35 NCG mice were randomly assigned to 5 groups. Briefly, 5 × 10^6^ MCF-7 cells were orthotopically injected into the mammary fat pad of 2 groups of mice, while 5 × 10^6^ MCF-7 PRKCQ-AS1 overexpressing (OE) cells, 5 × 10^6^ MCF-7 cells mixed with 1 × 10^6^ CAFs or NFs were orthotopically inoculated in the other 3 groups of mice, suspended in 100µL of PBS/Matrigel (1:1) (Yeasen, #40183ES). Twenty-five days post-injection, one MCF-7 control group received intraperitoneal injections of corn oil, while the other four groups were treated with tamoxifen citrate (Cayman, #11629) at a dose of 40 mg/kg every 2 days. Tumor size was measured every 5 days. After 45 days, mice were sacrificed and tumors were harvested for ISH, IHC and qPCR assays. Tumor volume was calculated using the formula: V = (0.5 × length × width^2)^ mm^3^.

### Statistical analysis

All experiments were performed in triplicate, and data are presented as means ± standard deviations (SDs). Statistical analyses were conducted using Student’s t-test, one-way analysis of variance (ANOVA), and two-way ANOVA. Kaplan-Meier analysis and Cox regression model were used to evaluate disease-free survival (DFS) and overall survival (OS). Statistical analyses were conducted using IBM SPSS Statistics software (version 21.0), R software (version 3.6.3), and GraphPad Prism (version 8.0). A two-side *P*-values < 0.05 was considered statistically significant.

## Results

### PRKCQ-AS1 predicts poor prognosis in tamoxifen-treated ER + breast cancer patients

**Fig. 1:**
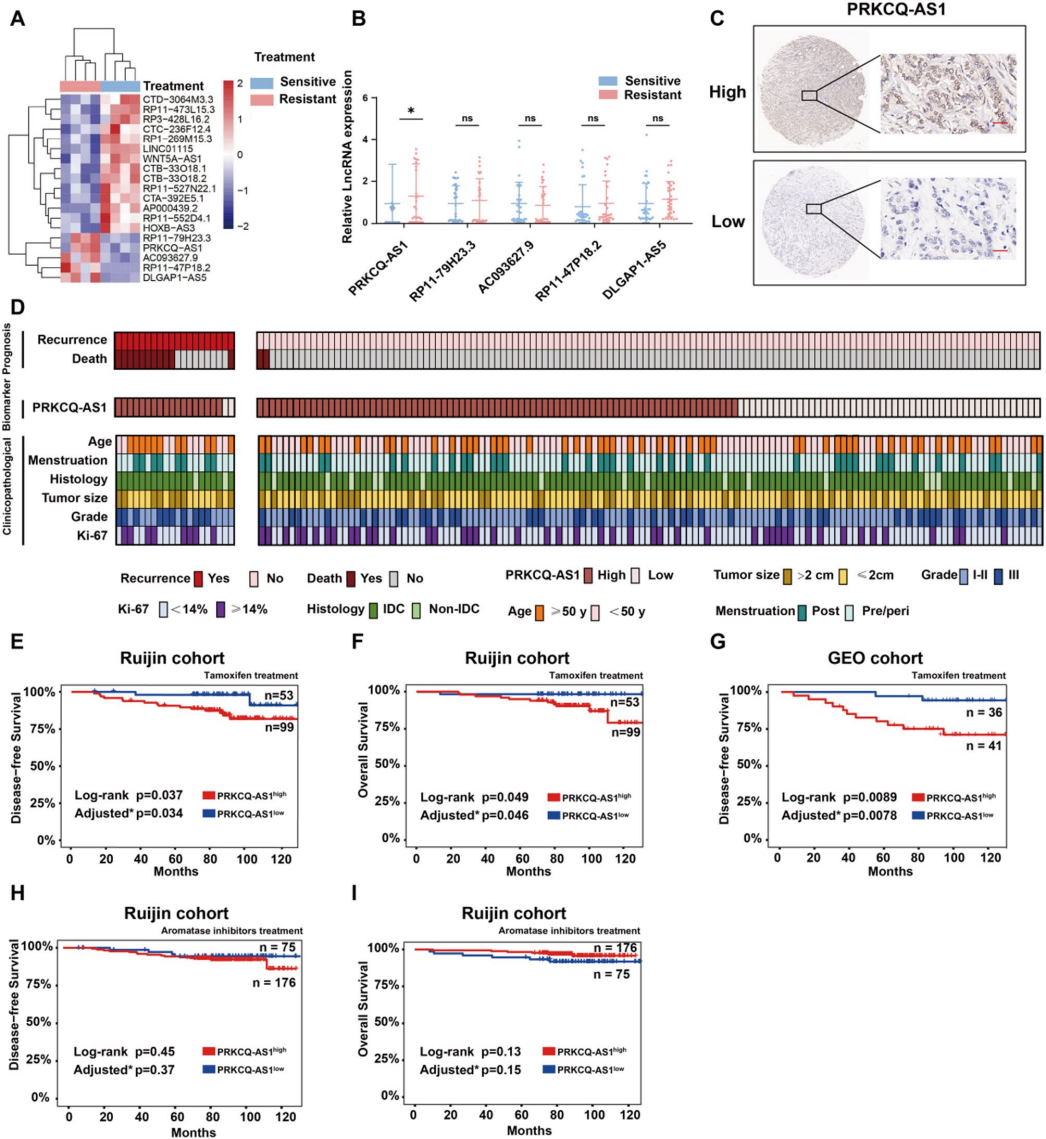
LncRNA PRKCQ-AS1 is associated with poor prognosis in tamoxifen-treated ER + breast cancer patients. **A.** Heatmap displaying differential transcriptomic expression from lncRNA-seq data in 4 tamoxifen-sensitive and 4 tamoxifen-resistant breast tumor tissues. **B**. qPCR analysis of the content of lncRNA PRKCQ-AS1, LINC02050, DLGAP1-AS5, LINC03015 and RP11-79H23.3 in 30 tamoxifen-sensitive and 30 tamoxifen-resistant breast tumor tissues. **C**. Representative images of ISH staining of PRKCQ-AS1 in tumor tissues from ER + tamoxifen-treated breast cancer patients in the Ruijin cohort. Scale bar: 50 μm. **D**. Integrated heatmap showing biomarkers (ISH of PRKCQ-AS1), clinicopathological features and prognosis in the Ruijin cohort consisting of 152 ER + breast cancer patients with tamoxifen treatment. **E-F**. Kaplan-Meier analysis of DFS and OS of PRKCQ-AS1 in ER + breast cancer patients treated with tamoxifen in the Ruijin cohort. Adjusted* *p* value is adjusted for age, menstruation, histology, tumor size, grade and ki-67. **G**. Kaplan-Meier analysis of DFS based on PRKCQ-AS1 expression in ER + breast cancer patients receiving tamoxifen treatment in the GEO cohort (GSE9195 database). Adjusted* *p* value is adjusted for age, menstruation, tumor size and grade. **H-I**. Kaplan-Meier analysis of DFS and OS based on PRKCQ-AS1 expression in ER + breast cancer patients receiving aromatase inhibitors treatment in the Ruijin cohort. Adjusted* *p* value is adjusted for age, menstruation, histology, tumor size, grade and ki-67. GEO: gene expression omnibus. *P < 0.05. Two-tailed Student’s t-test

To systematically identify differentially expressed genes between tamoxifen-sensitive and tamoxifen-resistant tumor tissues, bulk RNA-seq was performed on 4 tamoxifen-sensitive breast cancer samples and 4 tamoxifen-resistant ones. 5 up-regulated and 14 down-regulated lncRNAs in tamoxifen-resistant tumors were identified (Fig. 1A). Subsequent qPCR assays of 30 paired tumor samples from tamoxifen sensitive and resistant patients further confirmed that PRKCQ-AS1 was significantly up-regulated in tamoxifen-resistant tissues (Fig. 1B). To evaluate the clinical significance of PRKCQ-AS1 in tamoxifen resistance, ISH was performed on tissue microarrays from 152 tamoxifen-treated breast cancer patients in the Ruijin cohort (Fig. 1C). Clinicopathological and prognostic analysis of the cohort were shown in Fig. 1D. Prognostic analysis demonstrated that high PRKCQ-AS1 expression was associated with worse DFS and OS, regardless of clinicopathological variables (Fig. 1E-F). Consistent with these findings, analysis from the gene expression omnibus (GEO) cohort revealed a significant correlation between high PRKCQ-AS1 expression and poor DFS in tamoxifen-treated patients (Fig. 1G). However, no significant differences in DFS or OS were observed among patients treated with aromatase inhibitors, regardless of PRKCQ-AS1 expression levels (Fig. 1H-I).

### CAF-derived Exosomal PRKCQ-AS1 decreases tamoxifen sensitivity in ER + breast cancer cells

**Fig. 2:**
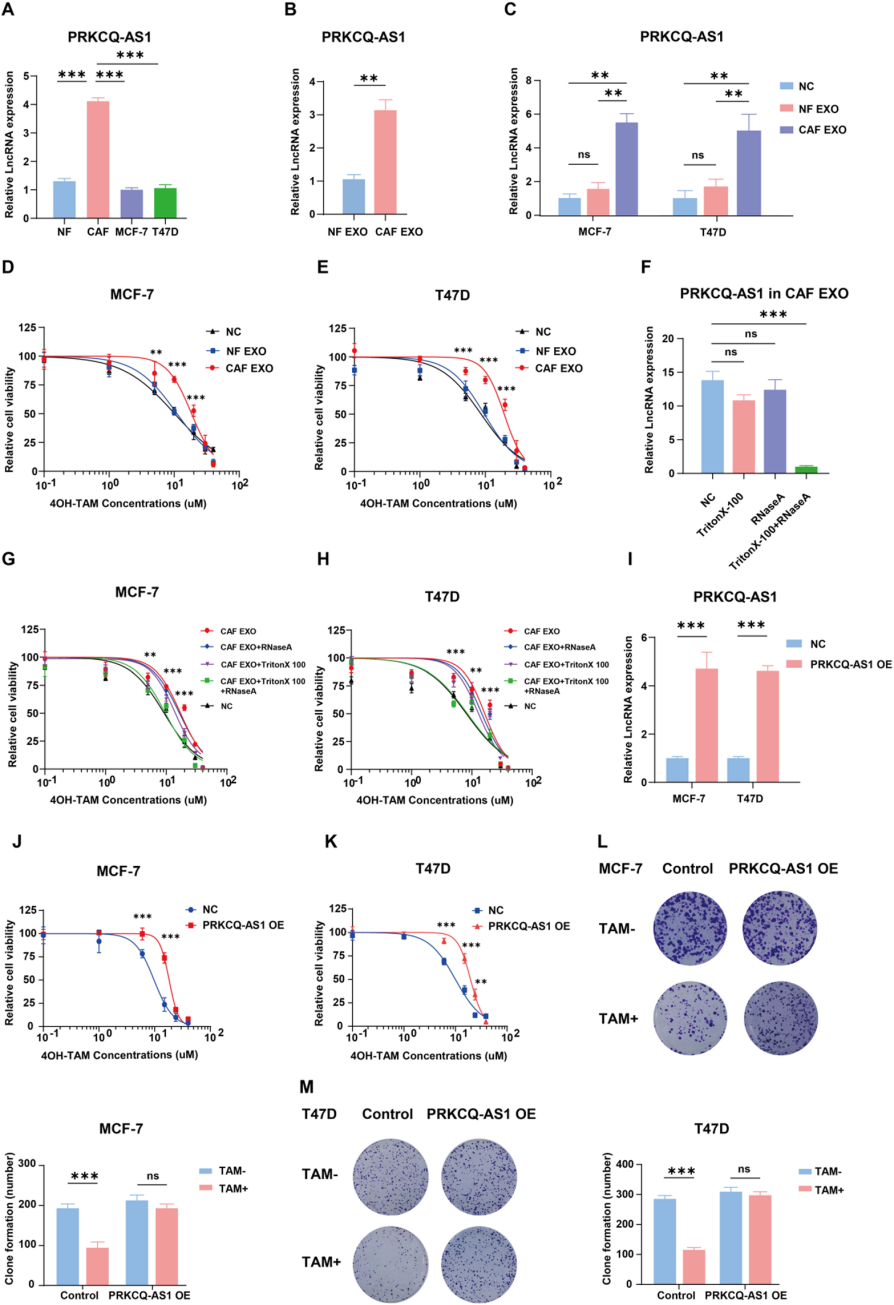
CAF-derived exosomal LncRNA PRKCQ-AS1 induces tamoxifen resistance of ER + breast cancer cells. **A**. qPCR analysis of lncRNA PRKCQ-AS1 in CAFs, NFs, MCF-7 and T47D. **B**. qPCR analysis of lncRNA PRKCQ-AS1 in CAF and NF-derived exosomes. **C**. qPCR analysis of lncRNA PRKCQ-AS1 in MCF-7 and T47D cells treated with CAF-derived exosomes, NF-derived exosomes or a negative control for 48 h. **D-E**. Cell titer glo analysis of MCF7 and T47D cells treated with a concentration gradient of tamoxifen in combination with CAF-derived exosomes, NF-derived exosomes or a negative control respectively for 72 h. **F**. qPCR analysis of PRKCQ-AS1 in CAF-derived exosomes treated with RNaseA (2 mg/ml), Triton X-100 (0.1%) or a combination of both for 20 min. **G-H**. Cell titer glo analysis of MCF7 and T47D cells treated with a concentration gradient of tamoxifen in combination with CAF-derived exosomes, CAF-derived exosomes treated with RNaseA (2 mg/ml) or Triton X-100 (0.1%) alone or both combined for 20 min, and a negative control for 72 h. **I**. qPCR analysis of PRKCQ-AS1 in the MCF-7 and MCF-7 PRKCQ-AS1 OE cells; T47D and T47D PRKCQ-AS1 OE cells. **J-K**. Cell titer glo analysis of MCF-7 and MCF-7 PRKCQ-AS1 OE cells; T47D and T47D PRKCQ-AS1 OE cells treated with a concentration gradient of tamoxifen for 72 h. **L-M**. Colony formation assays of MCF-7 and MCF-7 PRKCQ-AS1 OE cells; T47D and T47D PRKCQ-AS1 OE cells, with or without tamoxifen treatment (5µM) for 2 weeks. CAF: cancer-associated fibroblast; NF: normal fibroblast; EXO: exosome; NC: negative control; TAM: tamoxifen; OE: overexpressing. **P < 0.01, ***P < 0.001. Two-tailed Student’s t-test

To explore the resource of PRKCQ-AS1, we isolated seven CAFs from ER + breast tumors and three NFs from healthy controls and detected the content of PRKCQ-AS1 among CAFs, NFs, ER + breast cancer cells MCF-7 and T47D. qPCR assays demonstrated elevated expression levels of PRKCQ-AS1 in CAFs compared to NFs or tumor cells (Fig. 2A). Since one of the main ways that CAF affects the surrounding tumor cells is through exosomes, we extracted exosomes from the supernatant of CAF and NF culture media using ultracentrifugation. qPCR assays revealed higher expression of PRKCQ-AS1 in CAF-derived exosomes than NF-derived ones (Fig. 2B). Moreover, PRKCQ-AS1 in MCF7 and T47D cells upregulated after co-cultured with exosomes derived from CAFs (Fig. 2C) compared to cells treated with NF-derived exosomes or untreated controls and both MCF7 and T47D cells showed reduced tamoxifen sensitivity through this process (Fig. 2D-E). Notably, this effect was abrogated by Triton-X100 and RNaseA (Fig. 2F-H), indicating that lncRNA PRKCQ-AS1 within CAF-derived exosomes induced tamoxifen resistance in ER + breast cancer cells.

To assess the functional consequences of PRKCQ-AS1 overexpression, MCF-7 and T47D cells were infected with lentivirus to stably overexpress PRKCQ-AS1, as confirmed by qPCR (Fig. 2I). Compared to negative control (NC) groups, PRKCQ-AS1 overexpression (OE) cells exhibited reduced sensitivity to tamoxifen (Fig. 2J-K). Furthermore, PRKCQ-AS1 OE MCF-7 and T47D cells resisted tamoxifen-induced colony suppression (Fig. 2L-M). These findings suggest that lncRNA PRKCQ-AS1 contributes to reduced tamoxifen sensitivity in ER + breast cancer cells.

### PRKCQ-AS1 upregulates MKP1 expression to promote tamoxifen resistance

**Fig. 3:**
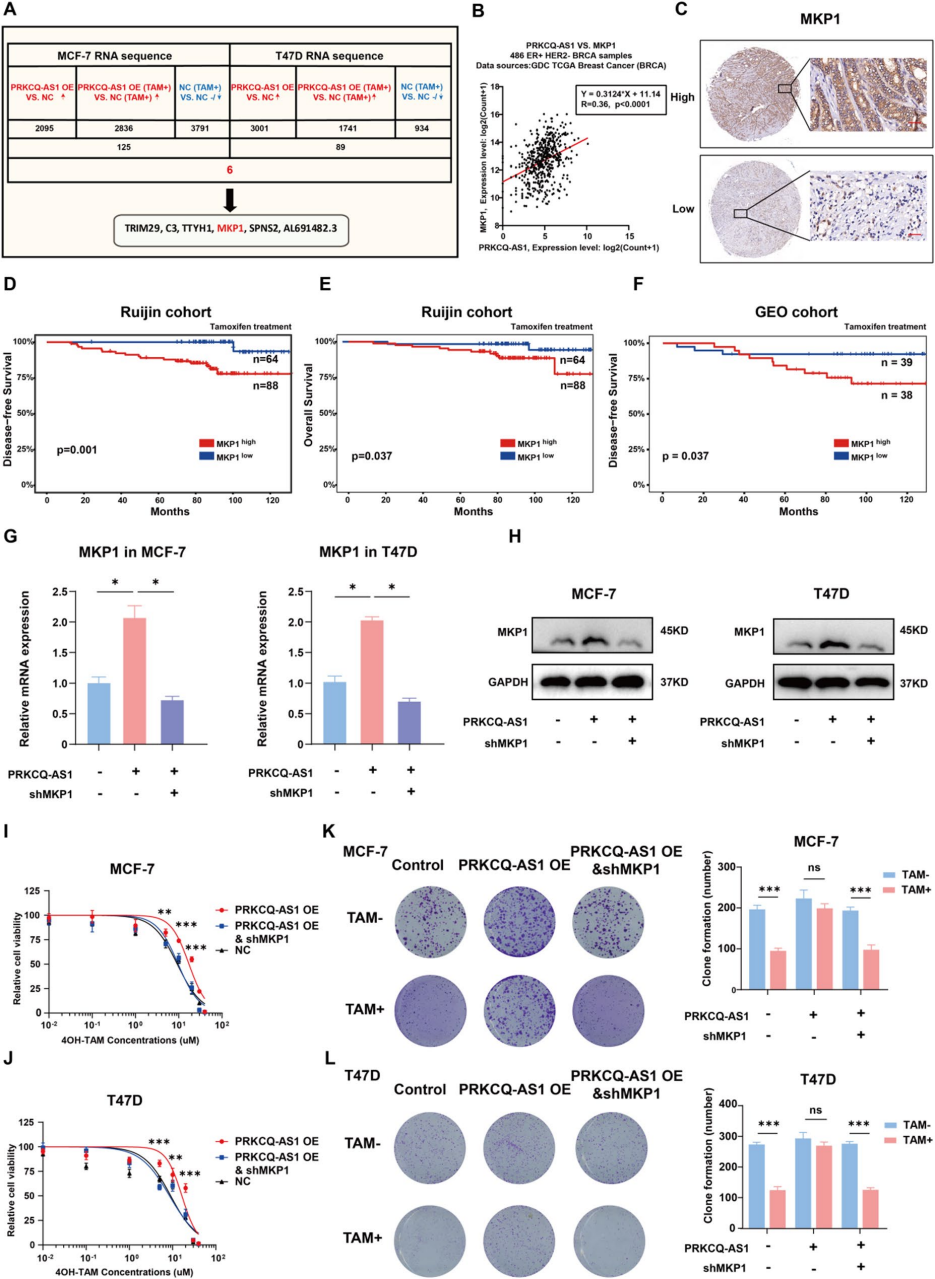
LncRNA PRKCQ-AS1 induces tamoxifen resistance via upregulating the expression of MKP1. **A**. Flow chart showing the identification of PRKCQ-AS1-regulated tamoxifen resistance related genes in MCF-7 and MCF-7 PRKCQ-AS1 OE cells; T47D and T47D PRKCQ-AS1 OE cells. **B**. Correlation analysis of PKRCQ-AS1 and MKP1 expression in 486 ER + HER2- breast tumor samples from the GDC TCGA Breast Cancer database. **C**. Representative images of IHC staining of MKP1 in tumor tissues from ER + tamoxifen-treated breast cancer patients in the Ruijin cohort. Scale bar,50 μm. **D**. Kaplan-Meier analysis of DFS of MKP1 expression in ER + breast cancer patients receiving tamoxifen treatment in the Ruijin cohort. **E**. Kaplan-Meier analysis of OS of MKP1 expression in ER + breast cancer patients receiving tamoxifen treatment in the Ruijin cohort. **F**. Kaplan-Meier analysis of DFS of MKP1 expression in ER + breast cancer patients receiving tamoxifen treatment in the GEO cohort (GSE9195 database). **G**. qPCR analysis of MKP1 expression in the MCF-7, MCF-7 PRKCQ-AS1 OE and MCF-7 PRKCQ-AS1 OE MKP1 knockdown cells; T47D, T47D PRKCQ-AS1 OE and T47D PRKCQ-AS1 OE MKP1 knockdown cells. **H**. Western blots analysis of MKP1 protein expression in the MCF-7, MCF-7 PRKCQ-AS1 OE and MCF-7 PRKCQ-AS1 OE MKP1 knockdown cells; T47D, T47D PRKCQ-AS1 OE and T47D PRKCQ-AS1 OE MKP1 knockdown cells. **I-J**. Cell titer glo analysis of MCF-7, MCF-7 PRKCQ-AS1 OE and MCF-7 PRKCQ-AS1 OE MKP1 knockdown cells; T47D, T47D PRKCQ-AS1 OE and T47D PRKCQ-AS1 OE MKP1 knockdown cells treated with a concentration gradient of tamoxifen for 72 h. **K-L**. Colony formation assay of MCF-7, PRKCQ-AS1 OE MCF-7 and PRKCQ-AS1 OE MKP1 knockdown MCF-7 cells; T47D, PRKCQ-AS1 OE T47D and PRKCQ-AS1 OE MKP1 knockdown T47D cells with or without tamoxifen treatment (5µM) for 2 weeks. TRIM29: tripartite motif-containing protein 29; C3: complement 3; TTYH1: tweety homolog 1; MKP1: mitogen-activated protein kinase phosphatase 1; SPNS2: spinster homolog 2; GDC: genomic data commons; TCGA: the cancer genome atlas; GEO: gene expression omnibus; GAPDH: glyceraldehyde-3-phosphate dehydrogenase; OE: overexpressing; NC: negative control; TAM: tamoxifen. *P < 0.05, ***P < 0.001. Two-tailed Student’s t-test

To identify genes regulated by PRKCQ-AS1 that may contribute to tamoxifen resistance, RNA-seq was performed on MCF7 and T47D cells with or without PRKCQ-AS1 overexpression, with or without tamoxifen treatment (Fig. 3A). Genes were selected based on the following criteria: upregulation in PRKCQ-AS1 OE cells compared with NCs, both with and without tamoxifen treatment, no upregulation in MCF-7 and T47D cells following tamoxifen treatment. Finally, six candidate genes were identified: MKP1, TRIM29, C3, TYTH1, SPNS2, and AL691482.3. Subsequent qPCR validation confirmed upregulation of MKP1 in MCF-7 and T47D PRKCQ-AS1 OE cells (Fig. S5A-B). Analysis of the Genomic Data Commons (GDC) the Cancer Genome Atlas (TCGA) Breast Cancer database indicated a positive correlation between PRKCQ-AS1 and MKP1 in 1,217 breast cancer samples (correlation coefficient = 0.2) (Fig. S6A), and a stronger correlation in 468 ER+/ human epidermal growth factor receptor2 (HER2)- breast cancer samples (correlation coefficient = 0.36) (Fig. 3B). However, no significant correlation was observed in 133 triple-negative breast cancer samples (Fig. S6B). Prognostic analysis using IHC staining on tissue microarrays from Ruijin cohort showed that MKP1 expression was associated with worse DFS and OS in 152 tamoxifen-treated but not in 251 aromatase inhibitors-treated ER + breast cancer patients (Fig. 3C-E, Fig. S4E, S4G). Consistently, GEO cohort analysis confirmed that high MKP1 expressions were remarkably associated with worse DFS in tamoxifen-treated breast cancer patients (Fig. 3F). qPCR and WB assays verified MKP1 upregulation in PRKCQ-AS1 OE cells (Fig. S6C-D).

To further validate the role of MKP1 in tamoxifen resistance, shRNA-mediated knockdown of MKP1 was performed in MCF-7 and T47D PRKCQ-AS1 OE cells, with knockdown efficiency confirmed via qPCR and WB (Fig. 3G-H). As a result, MKP1 knockdown restored tamoxifen sensitivity in PRKCQ-AS1 OE cells (Fig. 3I-L). Additionally, treatment with triptolide, an MKP1 inhibitor, reduced MKP1 expression in MCF7 and T47D cells co-cultured with CAF-derived exosomes (Fig. S7A-D) and significantly enhanced their response to tamoxifen (Fig. S7E-F). Collectively, these results demonstrated that PRKCQ-AS1 promotes tamoxifen resistance in ER + breast cancer cells through MKP1 upregulation and, targeting MKP1 may represent a potential therapeutic strategy to overcome PRKCQ-AS1-mediated tamoxifen resistance.

### PRKCQ-AS1 upregulates MKP1 expression via serving as a molecular sponge of miR-200a-3p

**Fig. 4:**
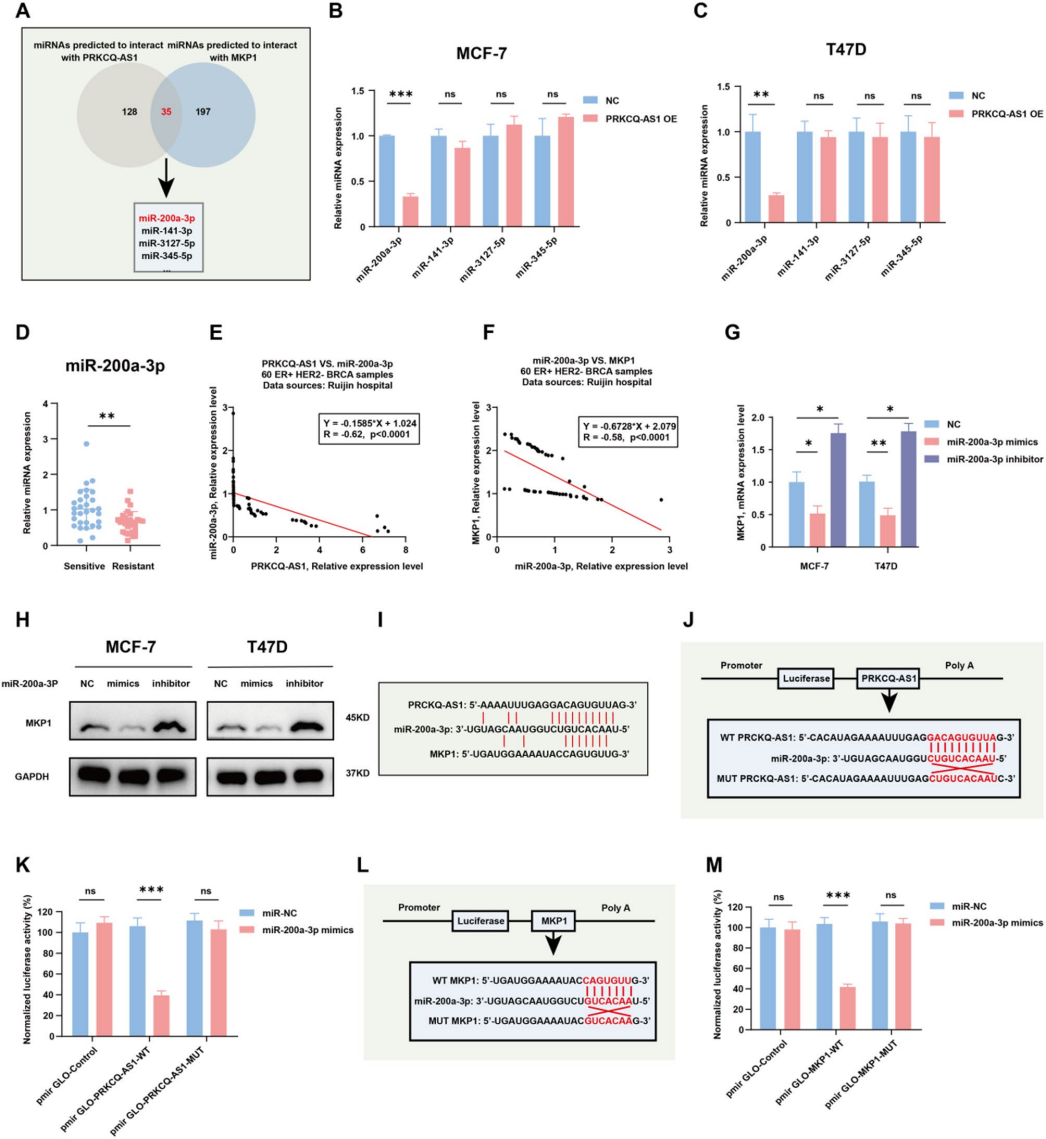
LncRNA PRKCQ-AS1 upregulates the expression of MKP1 via acting as a sponge of miR-200a-3p. **A.** Flow chart showing miRNAs predicted to both interact with PRKCQ-AS1 and MKP1 from the ENCORI online analysis database. **B-C**. qPCR analysis of miR-200a-3p, miR-141-3p, miR-3127-5p and miR-345-5p expression levels in MCF-7 and MCF-7 PRKCQ-AS1 OE cells; T47D and T47D PRKCQ-AS1 OE cells. **D.** qPCR analysis of the content of miR-200a-3p expression in 30 tamoxifen-sensitive and 30 tamoxifen-resistant breast tumor tissues. **E.** Correlation analysis of PRKCQ-AS1 and miR-200a-3p expression in 60 ER + HER2- breast tumor samples from Ruijin hospital. **F.** Correlation analysis of miR-200a-3p and MKP1 expression in 60 ER + HER2- breast tumor samples from Ruijin hospital. **G.** qPCR analysis of MKP1 expression in the MCF-7 and T47D cells transfected with miR-200a-3p mimics, miR-200a-3p inhibitor or negative control for 48 h. **H.** Western blots analysis of MKP1 protein expression in MCF-7 and T47D cells transfected with miR-200a-3p mimics, miR-200a-3p inhibitor or negative control for 48 h. **I.** Potential binding sites between PRKCQ-AS1 and miR-200a-3p; and between MKP1 and miR-200a-3p as identified in the ENCORI online database. **J-K.** Luciferase-reporter assay assessing the interactions between miR-200a-3p and its wild-type or mutant binding sites in the 3’untranslated region (3’-UTR) of PRKCQ-AS1 in HEK293T cells. **L-M**. Luciferase-reporter assay assessing the interactions between miR-200a-3p and its wild-type or mutant binding sites in the 3’-UTR of MKP1 in HEK293T cells. NC: negative control; GDC: genomic data commons; TCGA: the cancer genome atlas; MKP1: mitogen-activated protein kinase phosphatase 1; GAPDH: glyceraldehyde-3-phosphate dehydrogenase; WT: wild type; MUT: mutation; OE: overexpression. *P < 0.05, **P < 0.01, ***P < 0.001. Two-tailed Student’s t-test

LncRNAs could function as ceRNAs [21]. The sophisticated network of gene expressions is regulated by the interactions between lncRNAs, miRNAs, and mRNA targets [22]. To explore the mechanism by which PRKCQ-AS1 interacts with MKP1, we used the Encyclopedia of RNA Interactomes (ENCORI) online database to identify miRNAs that interact with both PRKCQ-AS1 and MKP1 (Fig. 4A). Among the 35 identified miRNAs, 4 miRNAs with the highest correlation coefficient were further validated using qPCR, showing that miR-200a-3p expression was significantly reduced in MCF-7 and T47D PRKCQ-AS1 OE cells (Fig. 4B-C). Analysis of the GDC TCGA Breast Cancer database showed a negative correlation between miR-200a-3p and PRKCQ-AS1 in 477 ER+/HER2- breast cancer samples (Fig. S8A), which was also observed between miR-200a-3p and MKP1 (Fig. S8B). Moreover, qPCR analysis of 30 tamoxifen-resistant tissues also revealed a significant downregulation of miR-200a-3p (Fig. 4D), which negatively correlated with both PRKCQ-AS1 and MKP1 (Fig. 4E-F). In addition, transfection of MCF-7 and T47D cells with miR-200a-3p mimics led to decreased MKP1 expression and increased tamoxifen sensitivity, whereas miR-200a-3p inhibition resulted in elevated MKP1 expression and enhanced tamoxifen resistance (Fig. 4G-H, Fig. S9A-B).

To further elucidate the interaction among PRKCQ-AS1, miR-200a-3P, and MKP1, we conducted dual-luciferase reporter assays as previously reported [23, 24]. Putative binding sites between PRKCQ-AS1 and miR‐200a-3p, as well as between miR‐200a-3p and MKP were predicted using the ENCORI database (Fig. 4I). The dual-luciferase reporter assays demonstrated that miR-200a-3p mimics significantly reduced the luciferase activity of HEK293T cells transfected with PRKCQ-AS1 wild-type but had no effect on PRKCQ-AS1 mutated constructs (Fig. 4J-K). Similarly, miR-200a-3p mimics reduced luciferase activity in cells expressing wild-type MKP1 but not in those with mutated MKP1 constructs (Fig. 4L-M). These findings indicate that PRKCQ-AS1 upregulates MKP1 by serving as a molecular sponge of miR-200a-3p, and inhibiting miR-200a-3p mitigate tamoxifen resistance in ER + breast cancer cells.

.

### MKP1 modulates MAPK/JNK pathway to suppress tumor apoptosis and promote tamoxifen resistance

**Fig. 5:**
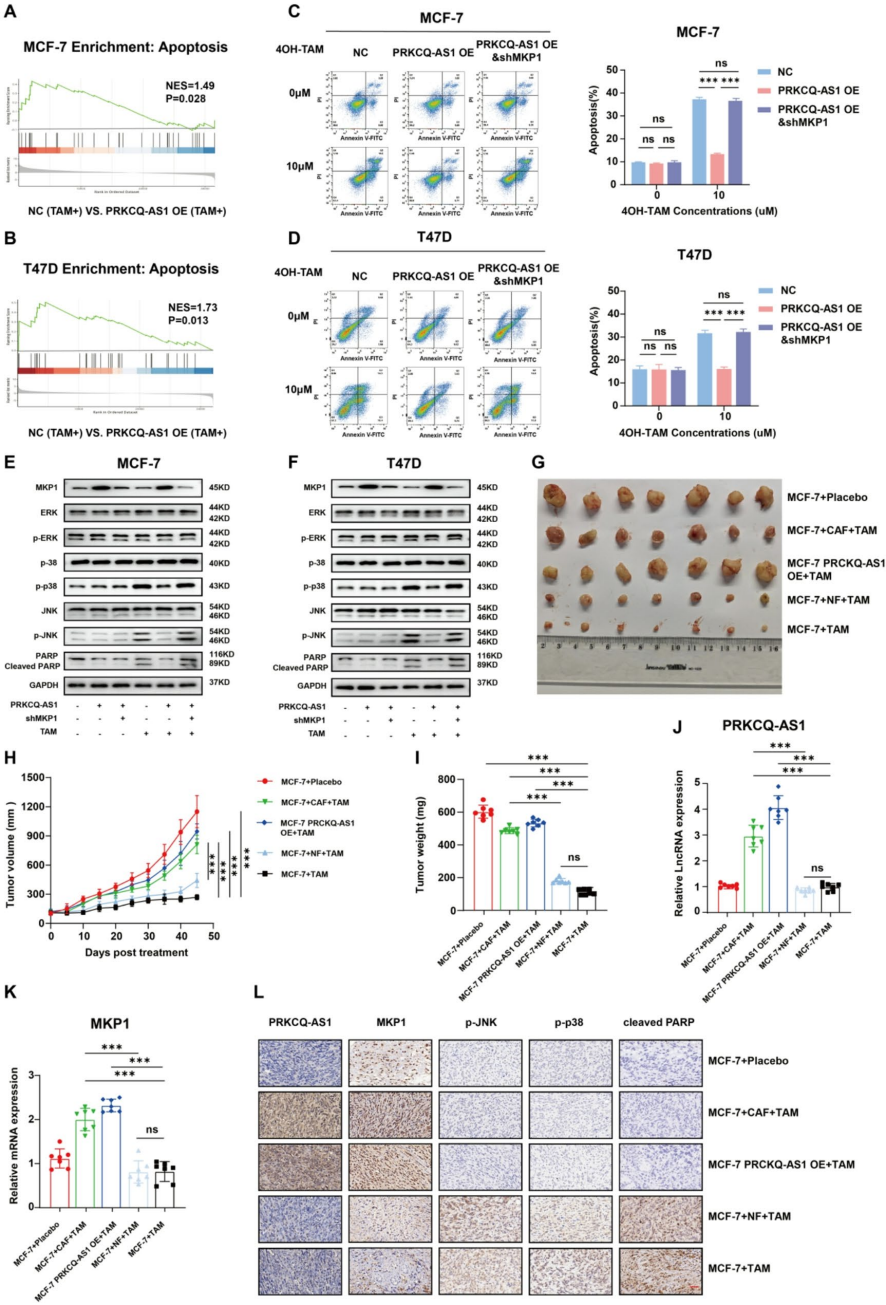
MKP1 upregulation inactivates MAPK/JNK pathway and reduces tamoxifen induced ER + breast cancer cells apoptosis. **A-B**. GSEA of the “Apoptosis_Multiple_Species_Cell_Growth_and_Death” gene set in MCF-7 and MCF-7 PRKCQ-AS1 OE cells treated with tamoxifen (10µM) for 48 h; T47D and T47D PRKCQ-AS1 OE cells treated with tamoxifen (10µM) for 48 h. **C-D**. Flow cytometry analysis of apoptosis in MCF-7, MCF-7 PRKCQ-AS1 OE and MCF-7 PRKCQ-AS1 OE MKP1 knockdown cells with or without tamoxifen treatment (10µM) for 48 h; T47D, T47D PRKCQ-AS1 OE and T47D PRKCQ-AS1 OE MKP1 knockdown cells with or without tamoxifen treatment (10µM) for 48 h. **E-F**. Western blots analysis of MKP1, ERK, p-ERK, p38, p-p38, JNK, p-JNK, PARP and cleaved PARP proteins in MCF-7, MCF-7 PRKCQ-AS1 OE and MCF-7 PRKCQ-AS1 OE MKP1 knockdown cells with or without tamoxifen treatment (10µM) for 48 h; T47D, T47D PRKCQ-AS1 OE and T47D PRKCQ-AS1 OE MKP1 knockdown cells with or without tamoxifen treatment (10µM) for 48 h. **G**. Representative images of excised tumors of NCG mice (*n* = 7 per group) with different orthotopic inoculation and treatment regimens. Tamoxifen (40 mg/kg*mice weight) every two days. **H**. Tumor growth curves of NCG mice (*n* = 7 per group) with different orthotopic inoculation and treatment regimens. **I**. Tumor weight of NCG mice (*n* = 7 per group) with different orthotopic inoculation and treatment regimens. **J**. qPCR analysis of PRKCQ-AS1 in excised tumors from NCG mice (*n* = 7 per group) with different orthotopic inoculation and treatment regimens. **K**. qPCR analysis of MKP1 in excised tumors from NCG mice (*n* = 7 per group) with different orthotopic inoculation and treatment regimens. **L**. ISH and IHC analysis of PRKCQ-AS1, MKP1, p-JNK, p-p38 and cleaved PARP expression in excised tumors from NCG mice (*n* = 7 per group) with different orthotopic inoculation and treatment regimens. Scale bar: 200 μm. NC: negative control; TAM: tamoxifen; MKP1: mitogen-activated protein kinase phosphatase 1; JNK: c-Jun N-terminal kinase; p38: p38 mitogen-activated protein kinase; ERK: extracellular signal-regulated kinase; p-p38: phosphorylated p38 mitogen-activated protein kinase; p-JNK: phosphorylated c-Jun N-terminal kinase; p-ERK: phosphorylated extracellular signal-regulated kinase; PARP: poly-ADP-ribose polymerase; GAPDH: glyceraldehyde-3-phosphate dehydrogenase; OE: overexpressing; CAF: cancer-associated fibroblast; NF: normal fibroblast. ***P < 0.001. Two-tailed Student’s t-test

To explore the mechanism by which PRKCQ-AS1 induces tamoxifen resistance in ER + breast cancer cells, GSEA of RNA-seq data from MCF-7 and T47D cells was performed. The apoptosis pathway was significantly downregulated in tamoxifen-treated MCF-7 and T47D PRKCQ-AS1 OE cells compared to NCs (Fig. 5A-B, Fig. S10A-B). Flow cytometry assays further confirmed a significantly lower apoptosis rate in tamoxifen-treated PRKCQ-AS1 OE cells compared to NCs, while knockdown of MKP1 in PRKCQ-AS1 OE cells significantly increased apoptosis rates (Fig. 5C-D). Previous studies have established that MKP1 dephosphorylates key proteins in the mitogen-activated protein kinase (MAPK) pathway, including c-Jun N-terminal kinase (JNK), p38 mitogen-activated protein kinase (p38), and extracellular signal-regulated kinase (ERK) [25]. Since tamoxifen induces cell growth arrest and apoptosis in ER + breast cancer cells by phosphorylating JNK and p38 [26], we examined the levels of phosphorylated p38 (p-p38) and JNK (p-JNK), as well as cleaved poly-ADP-ribose polymerase (PARP), in tamoxifen-treated cells. Increased levels of p-JNK, p-p38, and cleaved PARP were observed in tamoxifen-treated MCF-7 and T47D cells, but not in tamoxifen-treated PRKCQ-AS1-OE cells. MKP1 knockdown in PRKCQ-AS1-OE cells restored p-JNK, p-p38, and PARP cleavage (Fig. 5E-F). Furthermore, transfection with miR-200a-3p inhibitor resulted in decreased p-JNK and p-p38 levels and suppressed PARP cleavage in tamoxifen-treated MCF-7 and T47D cells (Fig. S9C-D). These results suggest that PRKCQ-AS1 induces tamoxifen resistance by upregulating MKP1 and activating MAPK/JNK pathway, which reduces tamoxifen treatment-induced apoptosis in ER + breast cancer cells.

### PRKCQ-AS1 induces tamoxifen resistance in vivo

To investigate the role of PRKCQ-AS1 in tamoxifen resistance in vivo, NCG mice were inoculated with MCF-7, MCF-7 PRKCQ-AS1 OE cells, MCF-7 cells mixed with CAFs or NFs, then were treated with tamoxifen or placebo. CAFs co-injection accelerated tumor growth than NFs despite tamoxifen, indicating that CAFs contribute to tamoxifen resistance in vivo (Fig. 5G-I). Moreover, tumors from MCF-7 PRKCQ-AS1-OE inoculated mice were larger than those from MCF-7 control mice following tamoxifen treatment, confirming that PRKCQ-AS1 reduces tamoxifen sensitivity (Fig. 5G-I). qPCR, ISH and IHC assays revealed elevated PRKCQ-AS1 and MKP1 expression in mice inoculated with MCF-7 PRKCQ-AS1 OE cells and MCF-7 cells mixed with CAFs (Fig. 5J-L). The expression levels of MKP1 were positively correlated with PRKCQ-AS1 (Fig. S11). Additionally, tamoxifen-treated mice with MCF-7 cells and MCF-7 cells mixed with NFs exhibited upregulated p-JNK, p-p38, and cleaved-PARP (Fig. 5L). These results indicate that PRKCQ-AS1 from CAF-derived exosomes promotes tamoxifen resistance in ER + breast cancer in vivo.

### PAX5 upregulates PRKCQ-AS1 expression in CAFs

**Fig. 6:**
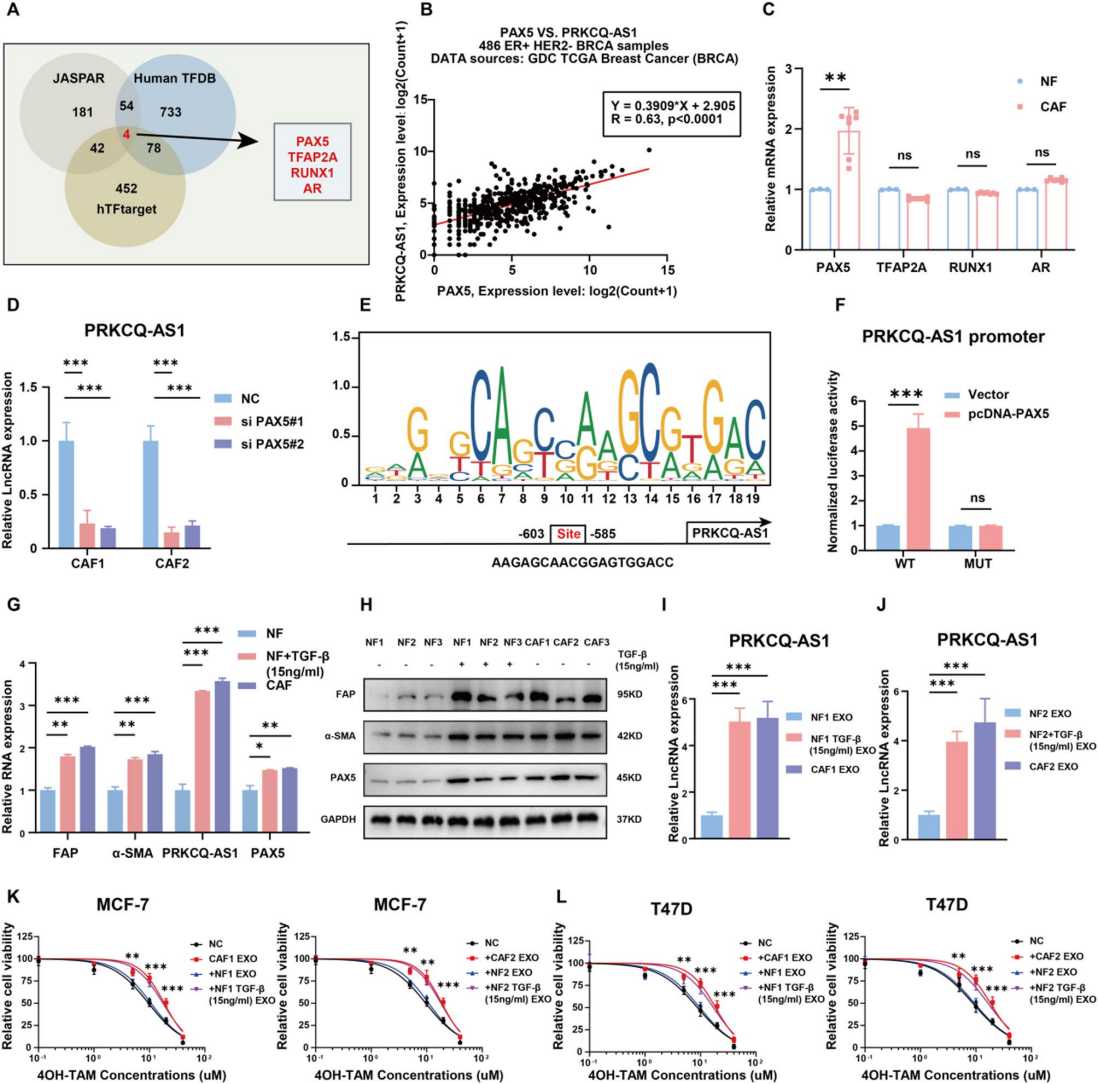
PAX5 upregulates LncRNA PRKCQ-AS1 expression and induces NFs transformation into CAFs. **A.** Flow chart showing transcription factors of PRKCQ-AS1 predicted using JASPAR, hTFtarget and human TFDB online databases. **B.** Correlation analysis of PRKCQ-AS1 and PAX5 expression in 486 ER + HER2- breast tumor samples from the GDC TCGA Breast Cancer database. **C.** qPCR analysis of PAX5, TFAP2A, RUNX1 and AR in 3 NFs and 7 CAFs. **D.** qPCR analysis of PRKCQ-AS1 in CAFs transfected with si PAX5#1, si PAX5#2 and negative control for 48 h. **E.** PAX5 DNA motif and predicted putative binding sites in the PRKCQ-AS1 promoter. **F.** Luciferase-reporter assay showing that PAX5 upregulates PRKCQ-AS1 promoter activity while mutations in promoter site eliminate this effect. **G.** qPCR analysis of FAP, α-SMA, PRKCQ-AS1, PAX5 in NFs, TGF-β (15ng/ml, 48 h) treated NFs and CAFs. **H.** Western blots detected the expression of FAP, α-SMA and PAX5 in NFs, TGF-β treated (15ng/ml, 48 h) NFs and CAFs. **I-J.** qPCR analysis of PRKCQ-AS1 levels in exosomes of NFs, TGF-β treated (15ng/ml, 48 h) NFs and CAFs. **K-L**. Cell titer glo analysis assessing the viability of MCF7 and T47D cells treated with a concentration gradient of tamoxifen in combination with exosomes from NFs, TGF-β treated (15ng/ml, 48 h) and CAFs and a negative control for 72 h. PAX5: paired box 5; TFAP2A: activating enhancer binding protein 2 α; RUNX1: runt-related transcription factor 1; AR: androgen receptor; GDC: genomic data commons; TCGA: the cancer genome atlas; ER: estrogen receptor; HER2: human epidermal growth factor receptor 2; CAF: cancer-associated fibroblast; NF: normal fibroblast; EXO: exosome; NC: negative control; TAM: tamoxifen; FAP: fibroblast activation protein; α-SMA: α-smooth muscle actin; TGF-β: transforming growth factor-β. *P < 0.05, **P < 0.01, ***P < 0.001. Two-tailed Student’s t-test

To explore the mechanisms underlying PRKCQ-AS1 upregulation in CAFs within the breast cancer TME, we utilized JASPAR, hTFtarget, and human TFDB databases to predict potential transcription factors in CAFs. Paired box 5 (PAX5), transcription factor activating enhancer binding protein 2 α (TFAP2A), runt-related transcription factor 1 (RUNX1) and androgen receptor (AR) were identified as candidate transcription factors with possible transcriptional activity for PRKCQ-AS1 (Fig. 6A). Analysis of the GDC TCGA Breast Cancer database revealed a strong positive correlation between PRKCQ-AS1 and PAX5 in 486 ER+/ HER2-negative breast cancer samples (Fig. 6B), whereas no significant correlation was observed with PRKCQ-AS1 and TFAP2A, RUNX1, or AR (Fig. S12A-C). Both qPCR and WB assays demonstrated significantly higher PAX5 expression in 7 CAFs compared to 3 NFs (Fig. 6C, Fig. S13A). Knockdown of PAX5 using siRNA resulted in decreased PRKCQ-AS1 expression in CAFs (Fig. 6D, Fig. S13B-C). To demonstrate the transcriptional regulation of PAX5 on PRKCQ-AS1, we searched DNA motif of PAX5 and predicted putative binding sites of PAX5 in PRKCQ-AS1 promoter (Fig. 6E). Luciferase reporter assay confirmed that PAX5 overexpression increased the PRKCQ-AS1 promoter’s luciferase activity, whereas luciferase activity remained unchanged following a mutation in the promoter site (Fig. 6F). All together, PAX5 acted as a transcription activator for PRKCQ-AS1.

Previous studies have demonstrated that NFs tend to transition into CAFs under the influence of transforming growth factor-β (TGF-β) within the TME [27]. In our experiments, treatment of NFs with TGF-β resulted in the upregulation of CAF markers FAP and α-SMA, as well as the transcription factor PAX5, and lncRNA PRKCQ-AS1 (Fig. 6G-H). qPCR assays further revealed a significant increase in PRKCQ-AS1 levels within the exosomes of TGF-β-treated NFs (Fig. 6I-J). Moreover, compared to exosomes derived from untreated NFs, those from TGF-β-treated NFs significantly decreased the sensitivity of MCF-7 and T47D cells to tamoxifen (Fig. 6K-L). These findings suggest that in the breast cancer TME, the upregulation of PAX5 during the transformation of NFs into CAFs enhances PRKCQ-AS1 expression, which subsequently mediates tamoxifen resistance in ER + breast cancer cells.

**Fig. 7:**
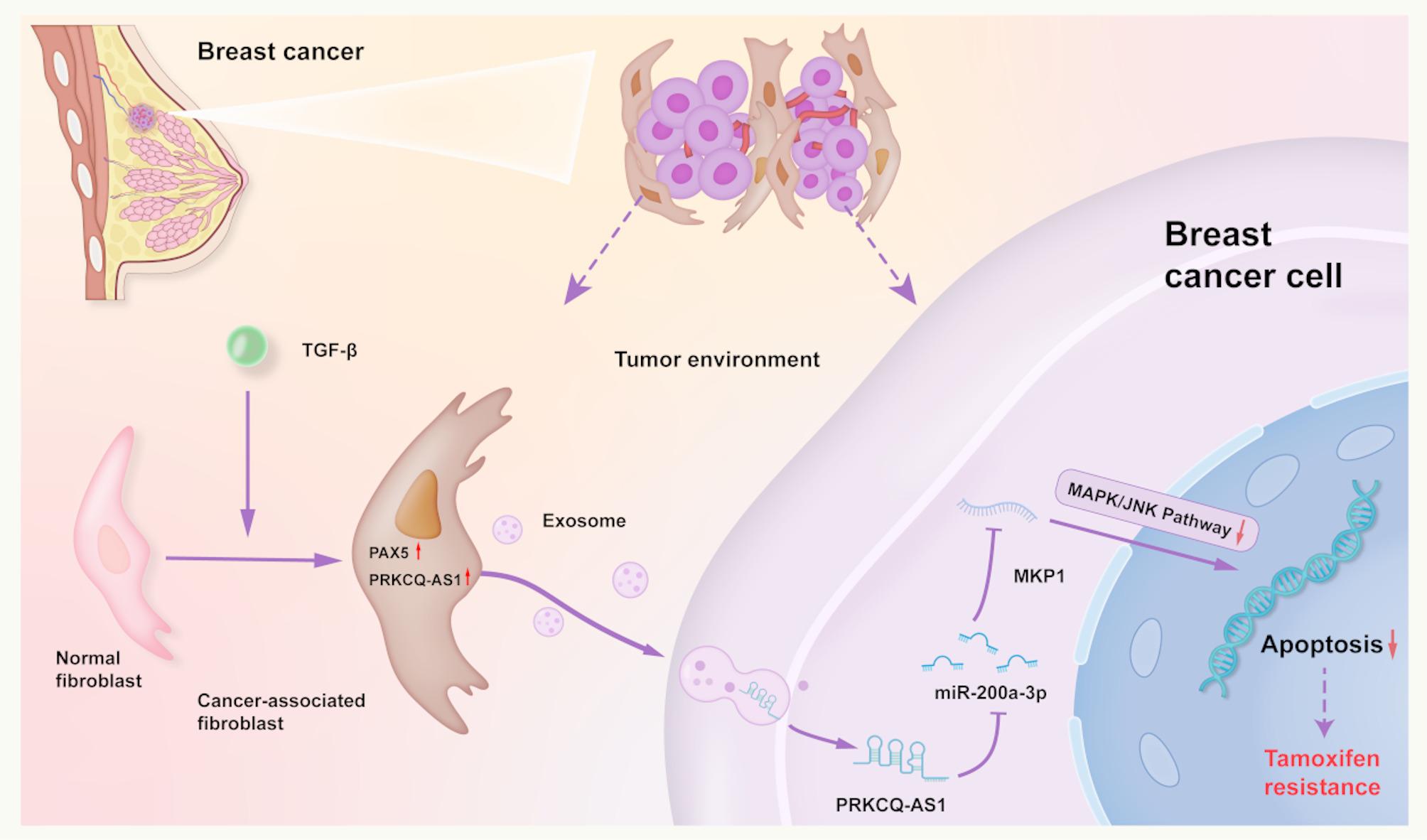
Summary diagram of CAFs contributing tamoxifen resistance in ER + breast cancer via exosomal lncRNA PRKCQ-AS1/miR-200a-3p/MKP1 axis. In breast cancer TME, NFs undergo transformation into CAFs under the influence of TGF-β, leading to the upregulation of PAX5 and PRKCQ-AS1. CAFs confer tamoxifen resistance to breast cancer cells by transferring exosomal lncRNA PRKCQ-AS1, which acts as a molecular sponge of miR-200a-3p, thereby upregulating MKP1 expression. Since tamoxifen primarily induces apoptosis in breast cancer cells, MKP1 counteracts this effect by reducing the phosphorylation of the MAPK/JNK pathway, ultimately attenuating apoptosis and promoting tamoxifen resistance

In conclusion, TGF-β promotes the conversion of NFs into CAFs in the breast cancer TME, accompanied by an increase in PAX5 and lncRNA PRKCQ-AS1 expression. CAFs transfer PRKCQ-AS1 to breast cancer cells via exosomes, where PRKCQ-AS1 upregulates MKP1 expression by serving as a molecular sponge of miR-200a-3p. This process reduces the phosphorylation levels of the MAPK/JNK pathway, thereby inhibiting tumor apoptosis and promoting resistance to tamoxifen treatment (Fig. 7).

## Discussion

Drug resistance has become severe challenges in cancer therapy [28–30]. Previous studies have suggested that TME played a key role in drug resistance [31]. As major component of breast cancer TME, CAFs-derived exosomal miR-18b promotes breast cancer invasion and metastasis by regulating TCEAL7 [19]. Additionally, podoplanin-positive CAFs contribute to trastuzumab resistance in HER2-positive breast cancer by secreting immunosuppressive factors, including indoleamine 2,3-dioxygenase 1 (IDO1) as well as tryptophan 2,3-dioxygenase 2 (TDO2) [32]. Furthermore, Fan et al. demonstrated that CAFs promote chemoresistance in breast cancer through the secretion of interleukin 6 and interleukin 8 [33]. However, the role of CAFs in endocrine therapy resistance in breast cancer remains largely unexplored. In this study, We identify CAF-derived exosomal lncRNA PRKCQ-AS1 as a novel mediator of tamoxifen resistance. PRKCQ-AS1 functions as a molecular sponge for miR-200a-3p, increasing MKP1 expression and inactivating JNK/p38-mediated apoptosis. Thus, PRKCQ-AS1 and MKP1 may serve as novel diagnostic biomarkers and therapeutic targets for ER + breast cancer patients undergoing tamoxifen treatment.

Tamoxifen resistance remains a significant clinical challenge [34]. CAF-derived exosomes have been shown to enhance adjacent tumor cell proliferation, metastasis, and drug resistance [9]. In this study, we primarily cultured CAFs to isolate and purify exosomes. Treatment of ER + breast cancer cells with CAF-derived exosomes resulted in reduced sensitivity to tamoxifen. Given that exosomes transport various proteins, DNA, RNA, and other biomolecules that influence drug response [35], we sought to identify the key factor mediating tamoxifen resistance in exosomes. Notably, treatment of CAF-derived exosomes with Triton X-100 and RNase A abolished tamoxifen resistance, indicating that RNA within these exosomes plays a crucial role in mediating this effect. LncRNAs, transcripts longer than 200 nucleotides lacking protein-coding ability [36, 37], have been shown to significantly contribute to drug resistance in breast cancer [38, 39]. Therefore, we explored the role of lncRNAs in tamoxifen resistance in ER + breast cancer. LncRNA PRKCQ-AS1, previously associated with colorectal cancer cells progression and migration, and paclitaxel resistance in triple negative breast cancer cells [40, 41], was found to be upregulated in 30 tamoxifen-resistant samples of bulk RNA-seq. Moreover, PRKCQ-AS1 expression was higher in CAFs-derived exosomes than in NF-derived exosomes. Both in vitro and in vivo assays confirmed that PRKCQ-AS1 overexpression led to tamoxifen resistance in ER + breast cancer cells. Furthermore, we found that PAX5 upregulates PRKCQ-AS1 expression during the transition of NFs into CAFs.

Recent studies have elucidated multiple mechanisms by which lncRNAs regulate tumor progression, including modulating chromatin state and methylation, protein and complex stability, or serving as a molecular sponge of specific miRNAs to regulate the expression of target mRNAs [35, 42–44]. For instance, lncRNA Lnc-408 promotes invasion and metastasis of breast cancer cells by regulating the miR-654-5p/LIM kinase 1 (LIMK1) axis [45], highlighting the intricate interplay among lncRNAs, miRNAs, and mRNAs in the breast cancer regulation. Our study also explores the underlying mechanism of PRKCQ-AS1 in modulating miR-200a-3p to upregulate the content of MKP1. MKP1, a dual-specificity phosphatase, recognizes and dephosphorylates the TXY amino acid motifs presented in MAPK family members, including JNK, p38, and ERK, with a higher affinity for JNK and p38 and less for ERK [25]. MAPK signaling pathway and, specifically, dephosphorylation of MAPK plays a key role in drug resistance and cancer metastasis [46–48]. Additionally, MKP1 has also been found to promote resistance to taxanes and anthracyclines in breast cancer [49]. Small et al. found that overexpression of MKP1 protects breast cancer cells from chemotherapy-mediated apoptosis when treated with doxorubicin and paclitaxel [50]. Given that Tamoxifen primarily induces apoptosis in breast cancer cells, reduced apoptosis is a key contributor to tamoxifen resistance [26]. Phosphorylation of JNK and p38 is essential for tamoxifen-induced apoptosis [51]. Besides, other study showed that MKP1 increased Bcl-2 expression, which might alleviate TAM-induced apoptosis [52]. In our study, tamoxifen-treated ER + breast cancer cells overexpressing PRKCQ-AS1 exhibited reduced apoptosis, accompanied by decreased levels of p-JNK, p-P38 and cleaved PARP. These findings suggest that PRKCQ-AS1 upregulates the expression of MKP1, leading to reduced tamoxifen-induced apoptosis and subsequent tamoxifen resistance in ER + breast cancer cells.

LncRNAs and their target mRNAs have been recognized as prognostic biomarkers in various cancers [53, 54]. Previous studies have reported that both PRKCQ-AS1 and MKP1 are associated with poor clinical outcomes in cancer patients. For instance, upregulation of PRKCQ-AS1 has been significantly linked to decreased survival in colorectal cancer patients [55]. High expression of MKP1 was related with shorter DFS in osteosarcoma [56]. Rincón et al. identified MKP1 as a potential predictive biomarker for a subset of breast cancer patients with poor clinical outcomes and reduced chemotherapy sensitivity [57]. In this study, we analyzed the expression of PRKCQ-AS1 and MKP1 in ER + breast cancer tissue microarrays and assessed patient prognosis. We found that tamoxifen-treated patients with high PRKCQ-AS1 or MKP1 expression were associated with decreased DFS and OS. Data from the GEO database further confirmed that high PRKCQ-AS1 or MKP1 expression in tumor tissue correlated with poor DFS in breast cancer patients undergoing tamoxifen treatment. However, in patients with ER + breast cancer receiving aromatase inhibitors treatment, PRKCQ-AS1 or MKP1 did not correlate with OS or DFS. Previous studies have reported that aromatase inhibitors have a lower apoptotic effect on tumor cells in ER + breast cancer patients compared to tamoxifen [58]. Given that PRKCQ-AS1 and MKP1 primarily mediate tamoxifen resistance by suppressing apoptosis, their prognostic value may be limited in patients treated with aromatase inhibitors. Therefore, PRKCQ-AS1 and MKP1 represent promising prognostic biomarkers for tamoxifen-treated ER + breast cancer patients.

## Conclusion

This study demonstrates that during the transformation of NFs into CAFs, PAX5 upregulates the expression of CAF-secreted exosomal lncRNA PRKCQ-AS1. PRKCQ-AS1 drives tamoxifen resistance in ER + breast cancer by upregulating MKP1 expression and reducing tamoxifen-induced apoptosis through serving as a molecular sponge of miR-200a-3p. Targeting CAF-derived exosomal PRKCQ-AS1 offers a promising therapeutic strategy to overcome tamoxifen treatment resistance in ER + breast cancer patients.

## Supplementary Information

Below is the link to the electronic supplementary material.

Supplementary Material 1

Supplementary Material 2

## Data Availability

GSE55561, GSE6532, and GSE9195 were obtained from the Gene Expression Omnibus (GEO) database (https://www.ncbi.nlm.nih.gov/geo/). GSE55561 contains gene expression data from PDX models with acquired resistance to tamoxifen, where the expression of CAF markers was analyzed in tamoxifen-sensitive and resistant PDX samples with two samples and five replicates each. Student’s t-test was used to analyze statistics in GSE55561. To further explore the relationship of CAF proportion and survival, Cibersortx was used to calculate the proportion of CAFs in tamoxifen-treated patients in GSE6532. GSE9195 includes transcriptomic data of breast cancer samples and survival outcomes of tamoxifen-treated patients, referring to GEO cohort. Data on content of lncRNAs, miRNAs and mRNAs (GDC TCGA BRCA) were obtained from TCGA database (https://www.cancer.gov/ccg/research/genome-sequencing/tcga). Binding sites for lncRNA and miRNA, and miRNA and mRNA were predicted on miRNA-Target part of ENCORI online analysis database (https://rnasysu.com/encori/index.php) with CLIP Data setting to “with or without data” “Degradome-Data >=0” and “pan-Cancer >= 0”. Potential transcription factors regulating PRKCQ-AS1 were predicted by JASPAR (https://jaspar.elixir.no/), hTFtarget (http://bioinfo.life.hust.edu.cn/hTFtarget#!/) and human TFDB (http://bioinfo.life/AnimalTFDB/). All other data used and analyzed in this study are available from the corresponding authors upon reasonable request.

## Abbreviations

α-SMA: α-smooth muscle actin
AGR2: Anterior gradient 2
AP-1: Activator protein-1
AR: Androgen receptor
CAF: Cancer-associated fibroblast
CD63: Cluster of differentiation 63
C3: Complement 3
ceRNAs: Competing endogenous RNAs
CI: Confidence interval
DAPI: 4,6-diamidino-2-phenylindole
DFS: Disease-free survival
DIG: Digoxigenin
ECL: Enhanced chemiluminescence
ENCORI: Encyclopedia of RNA Interactomes
ER +: Estrogen receptor positive
ERK: Extracellular signal-regulated kinase
FACS: Fluorescence activated cell sorting
FAP: Fibroblast activation protein
GDC: Genomic data commons
GEO: Gene expression omnibus
GSEA: Gene set enrichment analysis
HR: Hazard ratio
HER2: Human epidermal growth factor receptor 2
IDC: Invasive ductal carcinoma
IDO1: Indoleamine 2,3-dioxygenase 1
IHC: Immunohistochemistry
ISH: In situ hybridization
JNK: C-Jun N-terminal kinase
KLF12: Krueppel-like factor 12
LIMK1: LIM kinase 1
LncRNA: Long non-coding RNA
MAPK: Mitogen-activated protein kinase
MKP1: Mitogen-activated protein kinase phosphatase 1
NF: Normal fibroblast
NC: Negative control
OS: Overall survival
OE: Overexpressing
p38: p38 mitogen-activated protein kinase
PARP: Poly-ADP-ribose polymerase
PAX5: Paired box 5
p-ERK: Phosphorylated extracellular signal-regulated kinase
PI3K-AKT: Phosphatidylinositol3 kinase-RAC -serine/threonine-protein kinase
p-JNK: Phosphorylated c-Jun N-terminal kinase
PMSF: Phenylmethyl sulfonyl fluoride
p-p38: Phosphorylated p38 mitogen-activated protein kinase
PRKCQ-AS1: Protein kinase C theta antisense RNA 1
PVDF: Polyvinylidene difluoride
RIPA: Radio immunoprecipitation assay
ROC: Receiver operating characteristic
RUNX1: Runt-related transcription factor 1
shRNA: Short hairpin RNA
STMN1/EMT: Stathmin 1/ epithelial mesenchymal transition
SPNS2: Spinster homolog 2
TCGA: The cancer genome atlas
TDO2: Tryptophan 2,3-dioxygenase 2
TFAP2A: Transcription factor activating enhancer binding protein 2 α;
TGF-β: Transforming growth factor-β
TME: Tumor environment
TNBC: Triple negative breast cancer
TSG101: Tumor Susceptibility Gene 101
TRIM29: Tripartite motif-containing protein 29
TTYH1: Tweety homolog 1

## References

[1] Nolan E, Lindeman GJ. and J. E. Visvader. Deciphering Breast Cancer: From Biology to the Clinic. CELL. 2023;186(8):1708-28. 10.1016/j.cell.2023.01.040 36931265

[2] Sukocheva OA, Lukina E, Friedemann M, Menschikowski M, Hagelgans A, Gjumrakch Aliev. The crucial role of epigenetic regulation in breast cancer Anti-Estrogen resistance: current findings and future perspectives. Sem Cancer Biol. 2022;82:35–59. 10.1016/j.semcancer.2020.12.004 33301860

[3] Laws A, Punglia RS. Endocrine therapy for primary and secondary prevention after diagnosis of High-Risk breast lesions or preinvasive breast cancer. J Clin Oncol. 2023;41(17):3092–99. 37126767 10.1200/JCO.23.00455

[4] Guo H, Tan YQ, Huang X, Zhang S, Basappa B, Zhu T, Pandey V, Peter E. Lobie. Small molecule Inhibition of TFF3 overcomes Tamoxifen resistance and enhances taxane efficacy in ER + Mammary carcinoma. Cancer Lett. 2023;579:216443. 37858772 10.1016/j.canlet.2023.216443

[5] Szostakowska Małgorzata, Trębińska-Stryjewska A, Grzybowska EA, Fabisiewicz A. Resistance to endocrine therapy in breast cancer: molecular mechanisms and future goals. Breast Cancer Res Treat. 2019;173(3):489–97. 30382472 10.1007/s10549-018-5023-4 PMC6394602

[6] He H, Sinha I, Haldosen RFL-A, Zhao FYC, Dahlman-Wright K. C-Jun/AP-1 Overexpression Reprograms ERα Signaling Related to Tamoxifen Response in ERα-positive Breast Cancer. ONCOGENE. 2018;37(19):2586 – 600. 10.1038/s41388-018-0165-8 29467493

[7] de Visser KE, Joyce JA. The evolving tumor microenvironment: from cancer initiation to metastatic outgrowth. Cancer Cell. 2023;41(3):374–403. 36917948 10.1016/j.ccell.2023.02.016

[8] Qin Q, Yu R, Eriksson JE,. Cancer Lett. 2024;591:216859. 38615928 10.1016/j.canlet.2024.216859

[9] Hu D, Li Z, Zheng B, Lin X, Pan Y, Gong P, Zhuo W, Hu Y, Chen C, Chen L. Jichun zhou, and Linbo wang. Cancer-Associated fibroblasts in breast cancer: challenges and opportunities. Cancer Commun. 2022;42(5):401–34. 10.1002/cac2.12291 PMC9118050 35481621

[10] Namee N, Mc, Lorraine OD. Extracellular vesicles and Anti-Cancer drug resistance. Biochimica et biophysica ACTA-reviews on cancer. 2018;1870(2):123–36. 10.1016/j.bbcan.2018.07.003 30003999

[11] Maacha S, Bhat AA, Jimenez L, Raza A, Haris M, Uddin S. Extracellular Vesicles-Mediated intercellular communication: roles in the tumor microenvironment and Anti-Cancer drug resistance. Mol Cancer. 2019;18(1):55. 30925923 10.1186/s12943-019-0965-7 PMC6441157

[12] Gao Y, Li X, Zeng C, Liu C, Hao Q, Li W, Zhang K, Zhang W, Wang S, Zhao H, Li DFM, Zhang Y. Wei zhang, and Cun zhang. CD63 + cancer-Associated fibroblasts confer Tamoxifen resistance to breast cancer cells through Exosomal miR‐22. Adv Sci. 2020;7:21. 10.1002/advs.202002518 PMC7610308 33173749

[13] Sun J, Du R, Li X, Liu C, Wang D, He X, Li G, Zhang K, Wang S, Hao Q, Zhang Y, Li M, Gao Y, Zhang C. CD63(+) cancer-Associated fibroblasts confer CDK4/6 inhibitor resistance to breast cancer cells by Exosomal miR-20. Cancer Lett. 2024;588:216747. 38403110 10.1016/j.canlet.2024.216747

[14] Slack FJ, Arul M. Chinnaiyan. The Role of Non-Coding RNAs in Oncology. CELL. 2019;179(5):1033-55. 10.1016/j.cell.2019.10.017 PMC7347159 31730848

[15] Crudele F, Bianchi N, Reali E, Galasso M, Agnoletto C, Stefano Volinia. The network of Non-Coding RNAs and their molecular targets in breast cancer. Mol Cancer. 2020;19(1):61. 32188472 10.1186/s12943-020-01181-x PMC7079433

[16] Wu S, Lu J, Zhu H, Wu F, Mo Y, Xie L, Song C, Liu L, Xie X, Li Y. Huan lin, and Hailin tang. A novel axis of circKIF4A-miR-637-STAT3 promotes brain metastasis in Triple-Negative breast cancer. Cancer Lett. 2024;581:216508. 38029538 10.1016/j.canlet.2023.216508

[17] Zhang Y, Tan Y, Yuan J, Tang H, Zhang H, Tang Y, Xie Y, Wu L, Xie J, Xiao X, Li Y, Yanan Kong. CircLIFR-007 reduces liver metastasis via promoting hnRNPA1 nuclear export and YAP phosphorylation in breast cancer. Cancer Lett. 2024;592:216907. 38685451 10.1016/j.canlet.2024.216907

[18] Jiang M, Huang O, Xie Z, Wu S, Zhang X, Shen A, Liu H, Chen X, Wu J, Lou Y, Mao Y. Kan sun, Shudong hu, Meiyu geng, and Kunwei shen. A novel long Non-Coding RNA-ARA: adriamycin resistance associated. Biochem Pharmacol. 2014;87(2):254–83. 24184505 10.1016/j.bcp.2013.10.020

[19] Yan Z, Sheng Z, Zheng Y, Feng R, Shi QXL, Li H, Yin C, Luo H, Hao C, Wang W, Zhang B. Cancer-Associated Fibroblast-Derived Exosomal miR-18b promotes breast cancer invasion and metastasis by regulating TCEAL7. Cell Death Dis. 2021;12(12):1120. 34853307 10.1038/s41419-021-04409-w PMC8636636

[20] Shang C, Li Y, He T, Liao Y, Du Q, Wang P, Qiao J, Guo H. The prognostic miR-532-5p-correlated ceRNA-mediated lipid droplet accumulation drives nodal metastasis of cervical cancer. J Adv Res. 2022;37:169–84. 35499057 10.1016/j.jare.2021.09.009 PMC9040090

[21] Thomson DW, Marcel E. Dinger. Endogenous MicroRNA sponges: evidence and controversy. Nat Rev Genet. 2016;17(5):272–83. 27040487 10.1038/nrg.2016.20

[22] Qiao X, Ding Y, Altawil A, Yin Y, Wang Q, Wang W, Kang J. Roles of noncoding RNAs in chronic obstructive pulmonary disease. J Translational Intern Med. 2023;11(2):106–10. 10.2478/jtim-2023-0084 PMC10680378 38025954

[23] Zhou Y, Shao Y, Hu W, Zhang J, Shi Y, Kong X, Jiang J. A novel long noncoding RNA SP100-AS1 induces radioresistance of colorectal cancer via sponging miR-622 and stabilizing ATG3. Cell Death Differ. 2023;30(1):111–24. 35978049 10.1038/s41418-022-01049-1 PMC9883267

[24] Wang K, Zhou BLL-Y, Liu F, Zhou Q-Y, Liu C-Y, Fan Y-Y, Pei-Feng, Li. CARL LncRNA inhibits Anoxia-Induced mitochondrial fission and apoptosis in cardiomyocytes by impairing miR-539-dependent PHB2 downregulation. Nature Communications. 2014;5(1):3596. 24710105 10.1038/ncomms4596

[25] Haagenson KK. The role of MAP kinases and MAP kinase Phosphatase-1 in resistance to breast cancer treatment. Cancer Metastasis Rev. 2010;29(1):143–49. 20111893 10.1007/s10555-010-9208-5 PMC4063276

[26] García-Becerra Rocío, Santos N, Díaz L. Mechanisms of resistance to endocrine therapy in breast cancer: focus on signaling pathways, MiRNAs and genetically based resistance. Int J Mol Sci. 2013;14(1):108–45. 10.3390/ijms14010108 PMC3565254 23344024

[27] Chen Y, Zhu S, Liu T, Zhang S, Lu J, Fan W, Lin L, Xiang T, Yang J, Zhao X, Xi Y, Ma Y, Cheng G, Lin D, Wu C. Epithelial cells activate fibroblasts to promote esophageal cancer development. Cancer Cell. 2023;41(5):903–18. 36963399 10.1016/j.ccell.2023.03.001

[28] Ge W, Wang Y, Quan M, Mao T, Bischof EY, Xu H, Zhang X, Li S, Yue M, Ma J, Yang H, Wang L, Yu Z, Wang L, Jiujie Cui. Activation of the PI3K/AKT signaling pathway by ARNTL2 enhances cellular Glycolysis and sensitizes pancreatic adenocarcinoma to erlotinib. Mol Cancer. 2024;23(1):48. 38459558 10.1186/s12943-024-01965-5 PMC10921723

[29] Zhu C, Xie Y, Li Q, Zhang Z, Chen J, Zhang K, Xia X, Yu D, Chen D, Zhengyuan Yu, and, Chen J. CPSF6-mediated XBP1 3’UTR shortening attenuates Cisplatin-Induced ER stress and elevates Chemo-Resistance in lung adenocarcinoma. Drug Resist Updates. 2023;68:100933. 10.1016/j.drup.2023.100933 36821972

[30] Jiang M, Qi F, Zhang K, Zhang X, Ma J, Xia S, Chen L, Yu Z, Chen J, Chen D. MARCKSL1–2 reverses Docetaxel-Resistance of lung adenocarcinoma cells by recruiting SUZ12 to suppress HDAC1 and elevate miR-200b. Mol Cancer. 2022;21(1):150. 35864549 10.1186/s12943-022-01605-w PMC9306054

[31] Yang C, Deng X, Tang Y, Tang H. Chenglai Xia Nat Prod Reverse Cisplatin Resist Hypoxic Tumor Microenvironment Cancer Letters. 2024;598:217116. 10.1016/j.canlet.2024.217116 39002694

[32] Du R, Zhang X, Lu X, Ma X, Guo X, Shi C, Ren X, Ma X, He Y. Yuan gao, and Yunjiang liu. PDPN positive CAFs contribute to HER2 positive breast cancer resistance to trastuzumab by inhibiting Antibody-Dependent NK Cell-Mediated cytotoxicity. Drug Resist Updates. 2023;68:100947. 10.1016/j.drup.2023.100947 36812747

[33] Fan G, Yu B, Tang L, Zhu R, Chen J, Zhu Y, Huang H, Zhou L, Liu J, Wang W, Tao Z, Zhang F, Yu S, Lu X, Cao Y, Du S, Li H, Li J, Zhang J, Ren H, Gires O, Liu H, Wang X, Qin J, Wang H. TSPAN8(+) myofibroblastic cancer-Associated fibroblasts promote chemoresistance in patients with breast cancer. Sci Transl Med. 2024;16:741. 10.1126/scitranslmed.adj5705 38569015

[34] Waks AG, Winer EP,. JAMA-journal Am Med Association. 2019;321(3):288–300. 10.1001/jama.2018.19323 30667505

[35] Kalluri R, Valerie S. LeBleu. The Biology, Function, and Biomedical Applications of Exosomes. Science. 2020;367(6478). 10.1126/science.aau6977 PMC7717626 32029601

[36] Schmitt AM, Chang HY. Long Noncoding RNAs Cancer Pathways Cancer Cell. 2016;29(4):452–63. 27070700 10.1016/j.ccell.2016.03.010 PMC4831138

[37] Wang Jiahui, Hongcheng GE Zhengyuanyu. Non-Coding RNAs as potential mediators of resistance to lung cancer immunotherapy and chemotherapy. Oncol Res. 2025;33(5):1033–54. 40296912 10.32604/or.2024.058256 PMC12034021

[38] Raju GS, Rama E, Pavitra SS, Bandaru GL, Varaprasad GP, Nagaraju. Rama Rao malla, Yun Suk huh, and Young-Kyu han. HOTAIR: A potential metastatic, Drug-Resistant and prognostic regulator of breast cancer. Mol Cancer. 2023;22(1):65. 36997931 10.1186/s12943-023-01765-3 PMC10061914

[39] Zheng R, Jia J, Guan L, Yuan H, Liu K, Liu C, Ye W, Liao Y, Lin S, Huang O. Long Noncoding RNA lnc-LOC645166 Promotes Adriamycin Resistance Via NF-κB/GATA3 Axis in Breast Cancer. Aging (Albany, NY.). 2020;12(10):8893 – 912. 10.18632/aging.103012 PMC7288957 32461377

[40] Cui G, Zhao HL, Li L. Long noncoding RNA PRKCQ-AS1 promotes CRC cell proliferation and migration via modulating miR‐1287‐5p/YBX1 axis. J Cell Biochem. 2020;121(10):4166–75. 32619070 10.1002/jcb.29712

[41] Zheng S, Fu W, Huang Q, Zhou J, Lu K, Gu J, Ma R, Guo G. LncRNA PRKCQ-AS1 regulates Paclitaxel resistance in Triple‐Negative breast cancer cells throughmiR‐361‐5p/PIK3C3 mediated autophagy. Clinical and experimental pharmacology and physiology. 2023;50(6):431–42. 10.1111/1440-1681.13758 36732923

[42] Bartonicek N, Maag JLV, Dinger ME. Long noncoding RNAs in cancer: mechanisms of action and technological advancements. Mol Cancer. 2016;15(1):43. 27233618 10.1186/s12943-016-0530-6 PMC4884374

[43] Kong Y, Yang L, Wei W, Lyu N, Zou Y, Gao G, Ou X, Xie X, Tang H. CircPLK1 Sponges miR-296-5p to Facilitate Triple-Negative Breast Cancer Progression. Epigenomics. 2019;11(10):1163-76. 10.2217/epi-2019-0093 31337246

[44] Zou Y, Zheng S, Xiao W, Xie X, Yang A, Gao G, Xiong Z, Xue Z, Tang H, Xie X. CircRAD18 sponges miR-208a/3164 to promote Triple-Negative breast cancer progression through regulating IGF1 and FGF2 expression. Carcinogenesis. 2019;40(12):1469–79. 31001629 10.1093/carcin/bgz071

[45] Qiao Y, Jin T, Guan S, Cheng S, Wen S, Zeng H, Zhao M, Yang L, Wan X, Qiu Y, Li Q, Liu M, and Yixuan Hou. Long Non-Coding RNA Lnc-408 Promotes InvasionMetastasis of Breast Cancer Cell by Regulating LIMK1. Oncogene. 2021;40(24):4198 – 213. 10.1038/s41388-021-01845-y PMC8211561 34079084

[46] Reznik SE, Tiwari AK, Chavda V, Ashby CR Jr. The Delivery of N-myc Downstream-Regulated Gene 2 (NDRG2) Self-Amplifying mRNA Via Modified Lipid Nanoparticles as a Potential Treatment for Drug-Resistant and Metastatic Cancers. Med Rev (2021). 2024;4(3):235 – 38. 10.1515/mr-2024-0004 PMC11195423 38919399

[47] Wu L, Huang S, Tian W, Liu P, Xie Y, Qiu Y, Li X, Tang Y, Zheng S, Sun Y, Tang H, Du W, Tan W, Xie X. PIWI-interacting RNA-YBX1 inhibits proliferation and metastasis by the MAPK signaling pathway via YBX1 in Triple-Negative breast cancer. Cell Death Discovery. 2024;10(1):7. 38182573 10.1038/s41420-023-01771-w PMC10770055

[48] Ganesan K, Xu C, Wu S, Sui Y, Du B, Zhang J, Gao F, Chen J, Hailin Tang. Ononin inhibits tumor bone metastasis and osteoclastogenesis by targeting Mitogen-Activated protein kinase pathway in breast cancer. Research. 2024;7:553. 10.34133/research.0553 PMC11648741 39687715

[49] Shen J, Zhang Y, Yu H, Shen B, Liang Y, Jin R, Liu X, Shi L, Cai X. Role of DUSP1/MKP1 in tumorigenesis, tumor progression and therapy. Cancer Med. 2016;5(8):2061–68. 27227569 10.1002/cam4.772 PMC4884638

[50] Small GW, Yue Y, Shi, Linda S, Higgins, Robert Z. Orlowski. Mitogen-Activated protein kinase Phosphatase-1 is a mediator of breast cancer chemoresistance. Cancer Res. 2007;67(9):4459–66. 17483361 10.1158/0008-5472.CAN-06-2644

[51] Gu Y, Chen T, Lopez E, Wu W, Wang X, Cao J, Teng L. The therapeutic target of Estrogen Receptor-Alpha36 in Estrogen-Dependent tumors. J Translational Med. 2014;12:16. 10.1186/1479-5876-12-16 PMC3899443 24447535

[52] Rininger A, Dejesus C, Totten A, Wayland A, Halterman MW. MKP-1 antagonizes c/ebpbeta activity and lowers the apoptotic threshold after ischemic injury. Cell Death Differ. 2012;19(10):1634–43. 22522596 10.1038/cdd.2012.41 PMC3438493

[53] Wang Y, Ding X, He HHY, Lu Z, Wu P, Tian L, Xia T, Yin J, Yuan H, Shi G, Liu D, Jiang K, Miao Y. Long Non-Coding RNA Lnc‐PCTST predicts prognosis through inhibiting progression of pancreatic cancer by downregulation of TACC‐3. Int J Cancer. 2018;143(12):3143–54. 29978472 10.1002/ijc.31657

[54] Pei M, Li ZX, Zhao. Dysregulation of lnc-SNHG1 and miR-216b-5p correlate with chemoresistance and indicate poor prognosis of serous epithelial ovarian cancer. J Ovarian Res. 2020;13(1):144. 33302997 10.1186/s13048-020-00750-4 PMC7731520

[55] Shademan M, Salanghuch AN, Zare K, Zahedi M, Foroughi MA, Rezayat KA. Hooman Mosannen mozaffari, Kamran ghaffarzadegan, Ladan goshayeshi, and Hesam dehghani. Expression profile analysis of two antisense LncRNAs to improve prognosis prediction of colorectal adenocarcinoma. Cancer Cell Int. 2019;19(1):278. 31708689 10.1186/s12935-019-1000-1 PMC6836367

[56] Fan Meng-ke, Zhang Guo-chuan, Chen W, Qi Li-li, Xie Ming-fang, Zhang Yue-yao. Ling wang, and Qi zhang. Siglec-15 promotes tumor progression in osteosarcoma via DUSP1/MAPK pathway. Front Oncol. 2021;11:710689. 34336699 10.3389/fonc.2021.710689 PMC8322944

[57] Rincon R, Zazo S, Chamizo C, Manso R, Gonzalez-Alonso P, Martin-Aparicio E, Cristobal I, Canadas C, Perona R, Lluch A, Eroles P, Garcia-Foncillas J, Albanell J, Rovira A, Madoz-Gurpide J. Rojo. C-Jun N-Terminal kinase inactivation by Mitogen-Activated protein kinase phosphatase 1 determines resistance to taxanes and anthracyclines in breast cancer. Mol Cancer Ther. 2016;15(11):2780–90. 27599524 10.1158/1535-7163.MCT-15-0920

[58] Dowsett M, Smith IE, Ebbs SR, Dixon JM, Skene A, Griffith C, Boeddinghaus I, Salter J, Detre S, Hills M, Ashley S. Stephen francis, Geraldine walsh, and Roger a’hern. Proliferation and apoptosis as markers of benefit in neoadjuvant endocrine therapy of breast cancer. Clin Cancer Res. 2006;12(3):s1024–30. 10.1158/1078-0432.CCR-05-2127 16467120

