# Supplementary material for: Cancer-associated fibroblast promotes tamoxifen resistance in estrogen receptor positive breast cancer via exosomal LncRNA PRKCQ-AS1/miR-200a-3p/MKP1 axis-mediated apoptosis suppression
Source: J Exp Clin Cancer Res. 2025 Sep 30;44:274. doi: 10.1186/s13046-025-03529-x (PMC12487062; doi:10.1186/s13046-025-03529-x)
Supplement: Supplementary file 1 — Supplementary Material 1 [file 13046_2025_3529_MOESM1_ESM.pdf]

Supplementary tables

Table S1. The primers for plasmids construction.

ID	Sequence (5'-3')
PRKCQ-AS1-F	tagaagattctagagctagcAGCCAGGGACGCGCGCTCGGTC
PRKCQ-AS1-R	tccgatttaaattcgaattcAACCGTTTCTTGTTTAATTTCATTC
MKP1-shRNA-F	gatcGCTCTGTCAACGTGCGCTTCActcgagTGAAGCGCACGTTGACAGAGCttttttg
MKP1-shRNA-R	aattcaaaaaaGCTCTGTCAACGTGCGCTTCActcgagTGAAGCGCACGTTGACAGAGC

Table S2. Sequences of siRNAs, miRNA mimics and miRNA inhibitor.

RNA sequences	
si PAX5#1 sense	5'-UGUUUGAGAGGCAGCACUA-3'
si PAX5#1 antisense	5'-UAGUGCUGCCUCUCAAACA-3'
si PAX5#2 sense	5'-GGGGAGACUUGUUCACACA-3'
si PAX5#2 antisense	5'-UGUGUGAACAAGUCUCCCC-3'
miR-200a-3p mimcs sense	5'-UAACACUGUCUGGUAACGAUGU-3'
miR-200a-3p mimcs antisense	5'-ACAUCGUUACCAGACAGUGUUA-3'
miR-200a-3p inhibitor	5'-ACAUCGUUACCAGACAGUGUUA-3'

Table S3. The primers used to amplify mRNAs, lncRNAs and miRNAs.

ID	Sequence (5'-3')
PRKCQ-AS1-F	GTAGCCGCCCTATTCAGTTCACTTC
PRKCQ-AS1-R	CCTCCAAGCTGTGACTTCAACTAGG
RP11-79H23.3-F	GGCAGAAGACAAAGCAAGAGCAAAG
RP11-79H23.3-R	AAAGAAAGGAGGGAGGGAGGGAAG
AC093627.9-F	TAGGAGGTTCGTGAACGGATGGG
AC093627.9-R	GGGTGCCAGTTGTTTCTCCAAATTG
RP11-47P18.2-F	TGTTCTTCCGCCAATGTGTTCCTC
RP11-47P18.2-R	CCAATGACCCAGCCTGAAATCGG
DLGAP1-AS5-F	GGATGGGAATTACACAGCGAAATGC
DLGAP1-AS5-R	GAGCCTCCTCCCTTCCTCATGG
Human GAPDH-F	TGACTTCAACAGCGACACCCA
Hunma GAPDH-R	CACCCTGTTGCTGTAGCCAAA
Mouse GAPDH-F	AACGACCCCTTCATTGACCTCA
Mouse GAPDH-R	TGGTCATGAGTCCTTCCACGATACC
FAP-F	ATGAGCTTCCTCGTCCAATTCA
FAP-R	AGACCACCAGAGAGCATATTTTG
α-SMA1-F	GGCATTCACGAGACCACCTAC
α-SMA1-R	CGACATGACGTTGTTGGCATAC
MKP1-F	AGTACCCCACTCTACGATCAGG
MKP1-R	GAAGCGTGATACGCACTGC
miR-200a-3p-F	GCGCGTAACACTGTCTGGTAA
mIR-200a-3p-R	AGTGCAGGGTCCGAGGTATT
miR-3127-5P-F	CGATCAGGGCTTGTGGAAT
miR-3127-5P-R	AGTGCAGGGTCCGAGGTATT
miR-345-5P-F	GCGGCTGACTCCTAGTCCA
miR-345-5P-R	AGTGCAGGGTCCGAGGTATT
miR-141-3p-F	GCGCGTAACACTGTCTGGTAA
miR-141-3p-R	AGTGCAGGGTCCGAGGTATT
U6-F	CTCGCTTCGGCAGCACA
U6-R	AACGCTTCACGAATTTGCGT
PAX5-F	ACTTGCTCATCAAGGTGTCAG
PAX5-R	TCCTCCAATTACCCCAGGCTT
TFAP2A-F	AGGTCAATCTCCCTACACGAG
TFAP2A-R	GGAGTAAGGATCTTGCGACTGG
RUNX1-F	TGAGCTGAGAAATGCTACCGC
RUNX1-R	ACTTCGACCGACAAACCTGAG
AR-F	CCAGGGACCATGTTTTGCC
AR-R	CGAAGACGACAAGATGGACAA

Table S4. Primary antibodies for western blot, immunofluorescence and immunohistochemistry

Protein	Western blot concentration	Immunofluorescence concentration	Immunohistochemistry concentration	Specificity	Company Lot No.
GAPDH	1:2000	/	/	Rat anti-human	Proteintech Cat#80570-1-RR
FAP	1:1000	1:100	/	Rat anti-human	Abcam Cat#ab314456
α-SMA	1:1000	1:100	/	Mouse anti-human, mouse, Rat	Abcam Cat#ab7817
CD63	1:1000	/	/	Rat anti-human	Cell Signaling Technology Cat#13116
TSG101	1:2000	/	/	Rat anti-human, mouse	Proteintech Cat#28283-1-AP
MKP1	1:1000	/	1:50	Rat anti-human, mouse	Cell Signaling Technology Cat#35217
ERK1/2	1:1000	/	/	Rat anti-human, mouse, Rat	Cell Signaling Technology Cat#4695
p-ERK1/2	1:1000	/	/	Rat anti-human, mouse, Rat	Cell Signaling Technology Cat#4370
JNK	1:1000	/	/	Rat anti-human, mouse, Rat	Cell Signaling Technology Cat#9252
p-JNK	1:1000	/	1:50	Rat anti-human, mouse, Rat	Cell Signaling Technology Cat#4668
p38	1:1000	/	/	Rat anti-human, mouse, Rat	Cell Signaling Technology Cat#8690
p-p38	1:1000	/	1:400	Rat anti-human, mouse, Rat	Cell Signaling Technology Cat#4511
PARP	1:1000	/	1:100	Rat anti-human, mouse, Rat	Cell Signaling Technology Cat#9523
PAX5	1:1000	/	/	Rat anti-human, mouse, monkey	Cell Signaling Technology Cat#12709

Table S5. The sequences for the DIG-labeled probe of PRKCQ-AS1 in ISH.

ID	Sequence (5'-3')
PRKCQ-AS1	-DIG-UGGGCGACAAGUGGCUGGUAA-DIG-
