# Supplementary material for: Cancer-associated fibroblast promotes tamoxifen resistance in estrogen receptor positive breast cancer via exosomal LncRNA PRKCQ-AS1/miR-200a-3p/MKP1 axis-mediated apoptosis suppression
Source: J Exp Clin Cancer Res. 2025 Sep 30;44:274. doi: 10.1186/s13046-025-03529-x (PMC12487062; doi:10.1186/s13046-025-03529-x)
Supplement: Supplementary file 2 — Supplementary Material 2 [file 13046_2025_3529_MOESM2_ESM.pdf]

LncRNA PRKCQ-AS1 in cancer-associated fibroblast-derived exosome promotes tamoxifen resistance in estrogen receptor positive breast cancer via miR-200a-3p/MKP1 axis

Supplementary figures and figure legends:

Figure S1

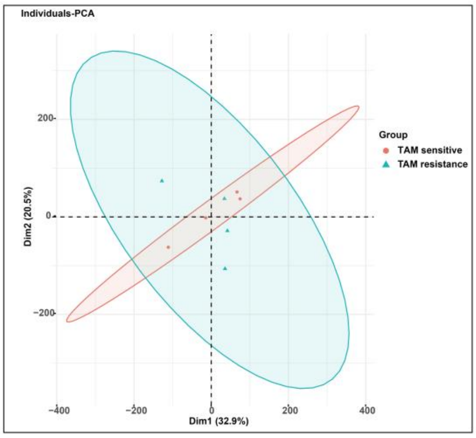

Figure S1. Principal component analysis plot of 4 tamoxifen-sensitive and 4 tamoxifen-resistant breast tumor tissues.

PCA: Principal component analysis; TAM: tamoxifen

Figure S2

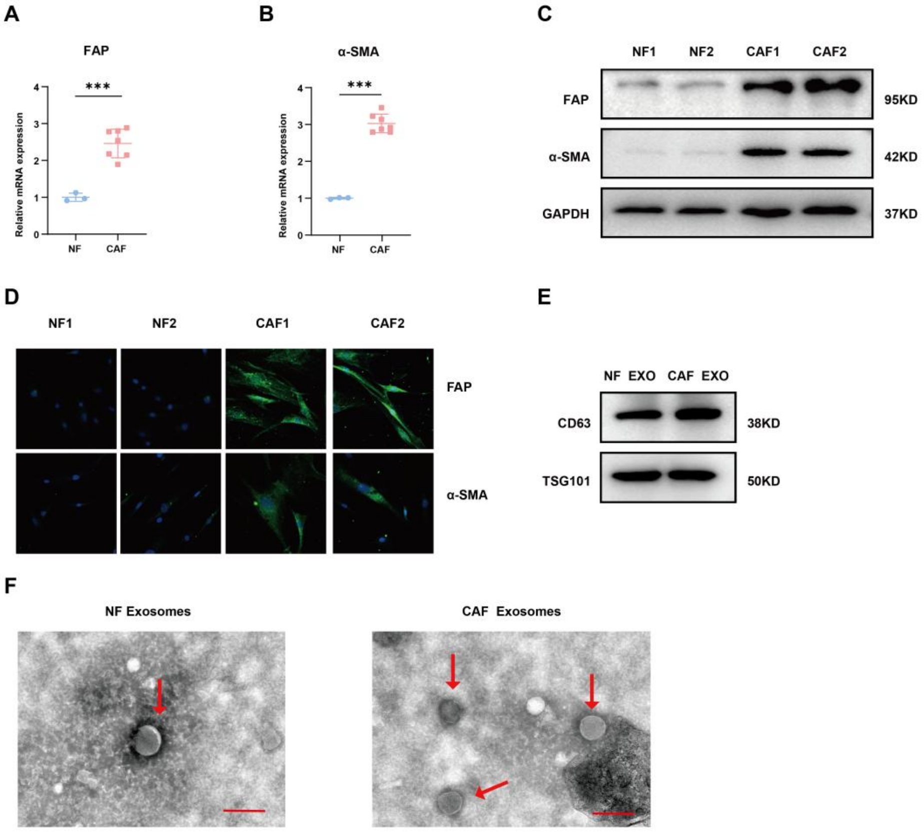

Figure S2. Identification of NFs, CAFs and their exosomes.

A-B. qPCR analysis of the content of FAP and α-SMA in isolated NFs and CAFs.

C. Western blots analysis of FAP and α-SMA protein in isolated NFs and CAFs.

D. Immunofluorescence analysis of the expression of α-SMA and FAP in isolated NFs and CAFs. Scale bar, 50μm.

E. Western blots analysis of exosome markers CD63 and TSG101 in NFs and CAFs derived exosomes.

F. Electron microscopic image showing isolated NFs and CAFs derived exosomes. Scale bar, 20nm.

FAP: fibroblast activation protein; α-SMA: α-smooth muscle actin; CAF: cancer-associated fibroblast; NF: normal fibroblast; EXO: exosome; GAPDH: glyceraldehyde-3-phosphate dehydrogenase; CD63: cluster of differentiation 63; TSG101: tumor Susceptibility Gene 101

***P<0.001. Two-tailed Student's t-test

Figure S3

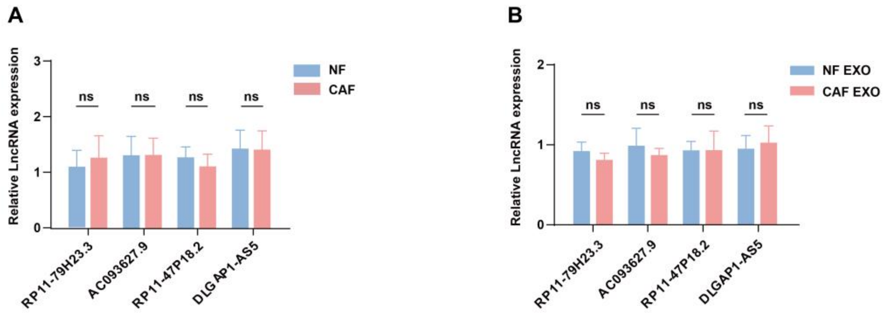

Figure S3. There exists no difference of other lncRNAs between CAFs and NFs; CAF and NF-derived exosomes.

A. qPCR analysis of lncRNA LINC02050, DLGAP1-AS5, LINC03015 and RP11-79H23.3 in CAFs and NFs.

B. qPCR analysis of lncRNA LINC02050, DLGAP1-AS5, LINC03015 and RP11-79H23.3 in CAF and NF-derived exosomes.

CAF: cancer-associated fibroblast; NF: normal fibroblast; EXO: exosome

Two-tailed Student's t-test

Figure S4

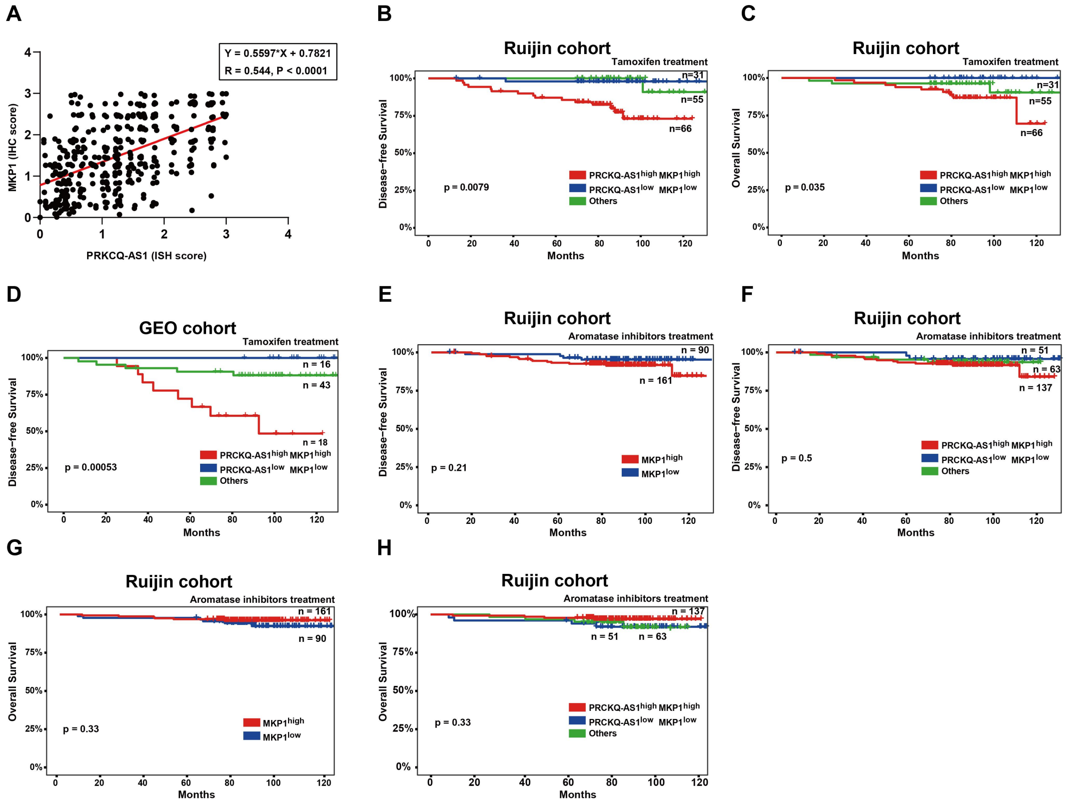

Figure S4. High PRKCQ-AS1 and MKP1 expression predicts poor prognosis in breast cancer patients with tamoxifen treatment.

A. Correlation between PRKCQ-AS1 and MKP1 expression from tumor tissues of ER+ breast cancer patients in Ruijin cohort determined by Pearson correlation analysis.

B. Kaplan-Meier analysis of disease-free survival of PRKCQ-AS1 and MKP1 in ER+ breast cancer patients with tamoxifen treatment from Ruijin cohort.

C. Kaplan-Meier analysis of overall survival of PRKCQ-AS1 and MKP1 in ER+ breast cancer patients with tamoxifen treatment from Ruijin cohort.

D. Kaplan-Meier analysis of overall survival of PRKCQ-AS1 and MKP1 in ER+ breast cancer patients with tamoxifen treatment from GEO cohort.

E-F. Kaplan-Meier analysis of disease-free survival of PRKCQ-AS1 or MKP1 in ER+ breast cancer patients with aromatase inhibitors treatment from Ruijin cohort.

G-H: Kaplan-Meier analysis of overall survival of PRKCQ-AS1 or MKP1 in ER+ breast cancer patients with aromatase inhibitors treatment from Ruijin cohort.

MKP1: mitogen-activated protein kinase phosphatase 1； ISH: in situ hybridization; IHC: immunohistochemistry

Figure S5

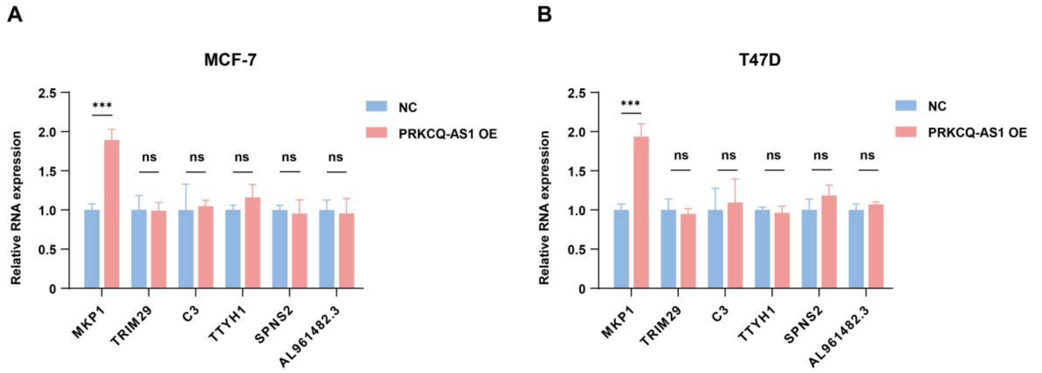

Figure S5. MKP1 upregulates in PRKCQ-AS1 OE ER+ breast cancer cells.

A-B. QPCR analysis of MKP1, TRIM29, C3, TTYH1, SPNS2, AL961482.3 in MCF-7 and MCF-7 PRKCQ-AS1 OE cells; T47D and T47D PRKCQ-AS1 OE cells.

OE: overexpression; TRIM29: tripartite motif-containing protein 29; C3: complement 3; TTYH1: tweety homolog 1; MKP1: mitogen-activated protein kinase phosphatase 1; SPNS2: spinster homolog 2

***P<0.001. Two-tailed Student's t-test

Figure S6

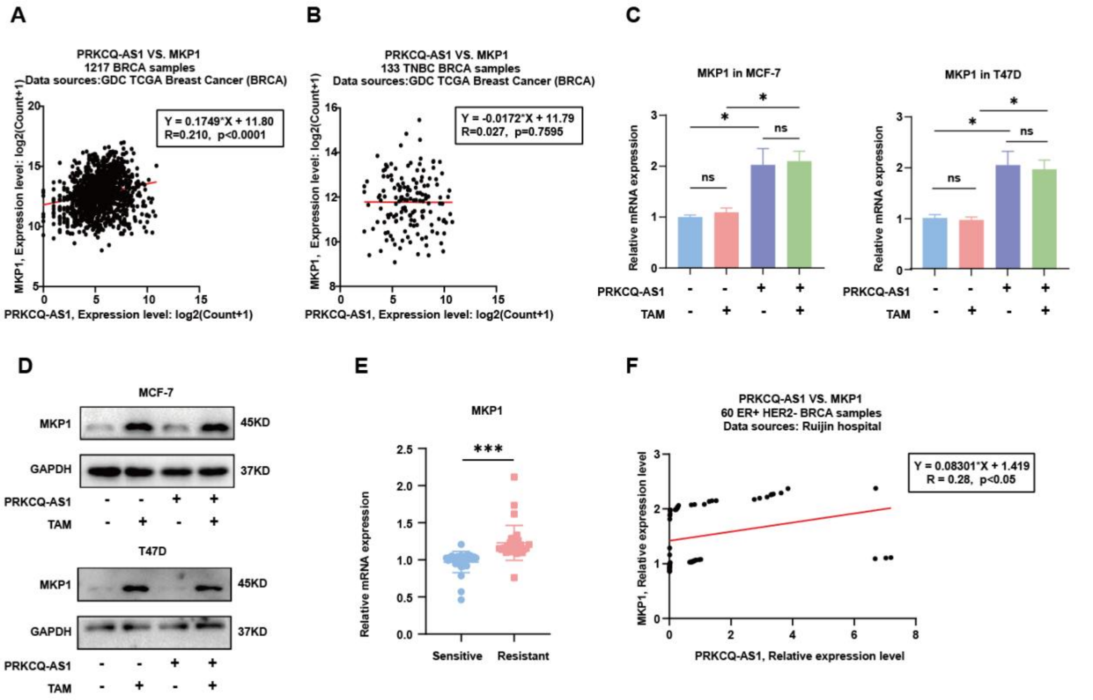

Figure S6. Correlation of expression of PKRCQ-AS1 and MKP1 in breast tumor samples from GDC TCGA Breast Cancer database.

A. Correlation of expression of PKRCQ-AS1 and MKP1 in 1218 breast tumor samples from GDC TCGA Breast Cancer database.

B. Correlation of expression of PKRCQ-AS1 and MKP1 in 147 TNBC breast tumor samples from GDC TCGA Breast Cancer database.

C. QPCR analysis of MKP1 in the MCF-7 and MCF-7 PRKCQ-AS1 OE cells with or without tamoxifen treatment (10μM) for 48h; T47D and T47D PRKCQ-AS1 OE cells with or without tamoxifen treatment (10μM) for 48h.

D. Western blots analysis of the expression of MKP1 in the MCF-7 and MCF-7 PRKCQ-AS1 OE cells with or without tamoxifen treatment (10μM) for 48h; T47D and T47D PRKCQ-AS1 OE cells with or without tamoxifen treatment (10μM) for 48h.

E. QPCR analysis of the content of MKP1 in 30 tamoxifen sensitive breast tumor tissues and 30 tamoxifen resistant breast tumor tissues.

F. Correlation of expression of PKRCQ-AS1 and MKP1 in 60 ER+ HER2- breast tumor samples from Ruijin hospital. *P<0.05, ***P<0.001. Two-tailed Student's t-test GDC: genomic data commons; TCGA: the cancer genome atlas; MKP1: mitogen-activated protein kinase phosphatase 1; ER: estrogen receptor; HER2: human epidermal growth factor receptor 2

*P<0.05, ***P<0.001. Two-tailed Student's t-test.

Figure S7

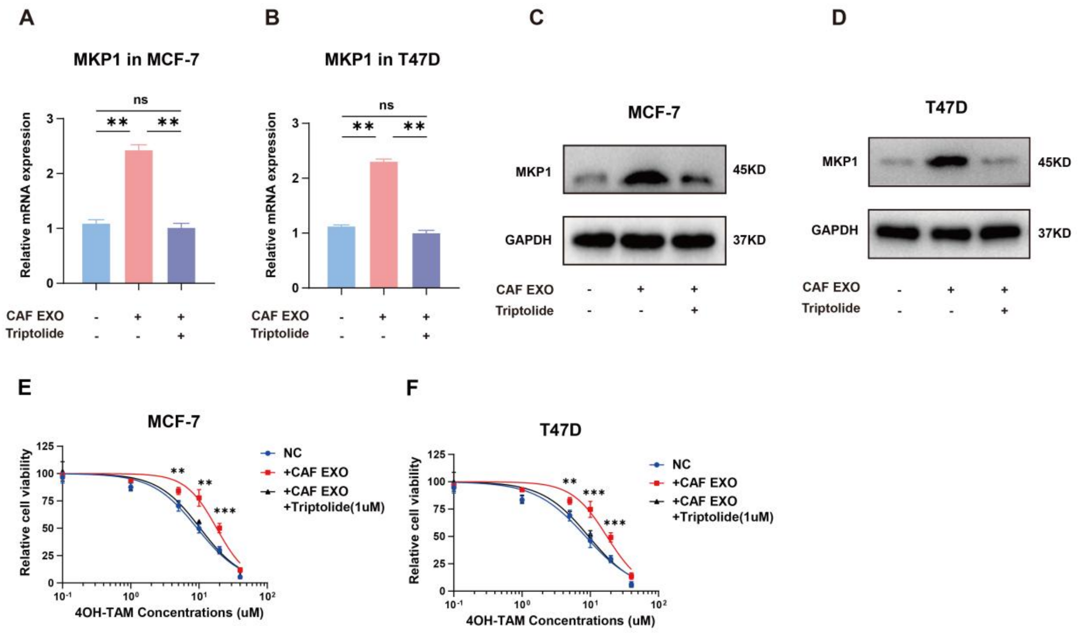

Figure S7. Triptolide sensitives CAF-derived exosomes induced tamoxifen resistance of ER+ breast cancer cells.

A-B. qPCR analysis of MKP1 in the MCF-7 and T47D cells treated with CAF-derived exosomes, CAF-derived exosomes and triptolide (1μM), and negative control for 48h.

C-D. Western blots detected the expression of MKP1 in the MCF-7 and T47D cells treated with CAF-derived exosomes, CAF-derived exosomes and triptolide (1μM), and negative control for 48h.

E-F. Cell titer glo analysis of MCF7 and T47D cells treated with a concentration gradient of tamoxifen combined with CAF-derived exosomes, CAF-derived exosomes and triptolide (1μM), and negative control for 72h. **P<0.01. Two-tailed Student's t-test

CAF: cancer-associated fibroblast; EXO: exosome; MKP1: mitogen-activated protein kinase phosphatase 1; GAPDH: glyceraldehyde-3-phosphate dehydrogenase

**P<0.01. Two-tailed Student's t-test.

Figure S8

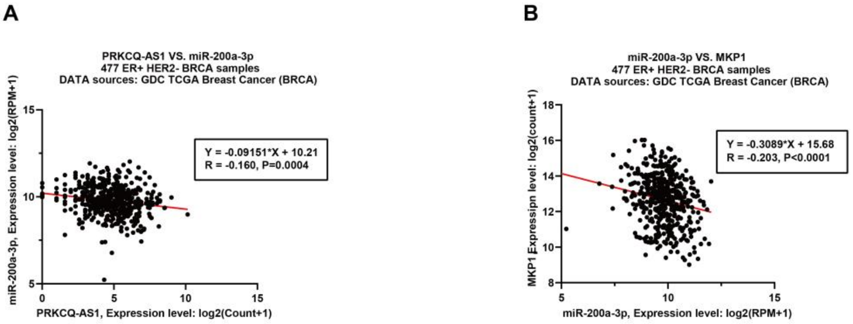

Figure S8. Correlation of expression of PKRCQ-AS1 and miR-200a-3p, miR-200a-3p and MKP1.

A. Correlation of expression of PKRCQ-AS1 and miR-200a-3p in 477 ER+ HER2- breast tumor samples from GDC TCGA Breast Cancer database.

B. Correlation of expression of miR-200a-3p and MKP1 in 477 ER+ HER2- breast tumor samples from GDC TCGA Breast Cancer database.

MKP1: mitogen-activated protein kinase phosphatase 1; GDC: genomic data commons; TCGA: the cancer genome atlas; ER: estrogen receptor; HER2: human epidermal growth factor receptor 2

Figure S9

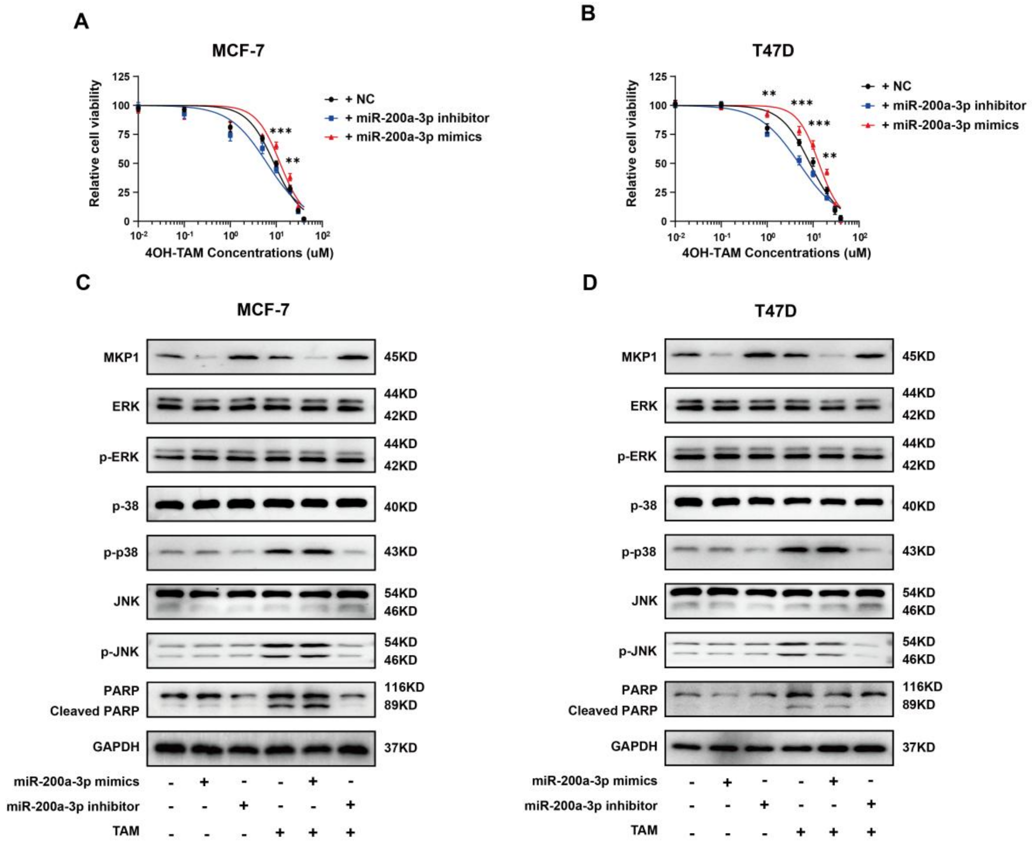

Figure S9. MiR-200a-3p increases tamoxifen sensitivity of ER+ breast cancer cells via activating MAPK/JNK pathway.

A-B. Cell titer glo analysis of MCF7 and T47D cells treated with a concentration gradient of tamoxifen and transfected with miR-200a-3p mimics, miR-200a-3p inhibitor and negative control for 72h.

C-D. Western blots analysis of MKP1, ERK, p-ERK, p-38, p-p38, JNK, p-JNK, PARP and cleaved PARP proteins in MCF-7 and T47D transfected with miR-200a-3p mimics, miR-200a-3p inhibitor and negative control, and treated with or without tamoxifen treatment (10μM) for 48h.

NC: negative control; TAM: tamoxifen; MKP1: mitogen-activated protein kinase phosphatase 1; JNK: c-Jun N-terminal kinase; p38: p38 mitogen-activated protein kinase; ERK: extracellular signal-regulated kinase; p-p38: phosphorylated p38mitogen-activated protein kinase; p-JNK: phosphorylated c-Jun N-terminal kinase; p-ERK: phosphorylated extracellular signal-regulated kinase; PARP:

poly-ADP-ribose polymerase; GAPDH: glyceraldehyde-3-phosphate dehydrogenase

Figure S10

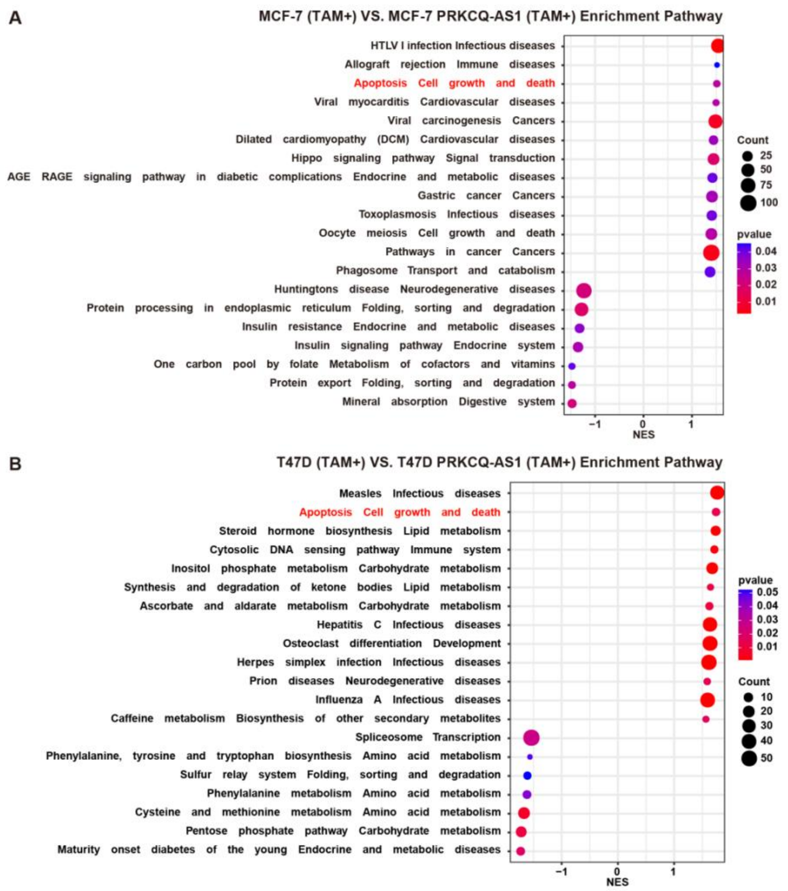

Figure S10. GSEA pathways for MCF-7 (TAM+) VS. MCF-7 PRKCQ-AS1 (TAM+) (A) and T47D (TAM+) VS. T47D PRKCQ-AS1 (TAM+) (B).

TAM: tamoxifen; NES: normalized enrichment score

Figure S11

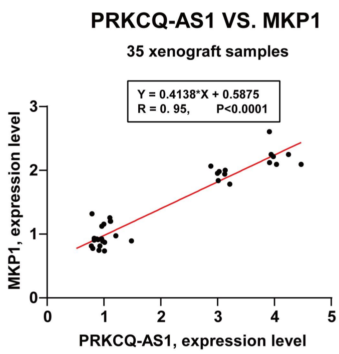

Figure S11. Expression levels of MKP1 were positively correlated with PRKCQ-AS1 in 35 excised tumors of NCG mice.

Correlation between PRKCQ-AS1 and MKP1 contents from excised tumors of 35 NCG mice determined by Pearson correlation analysis.

MKP1: mitogen-activated protein kinase phosphatase 1

Figure S12

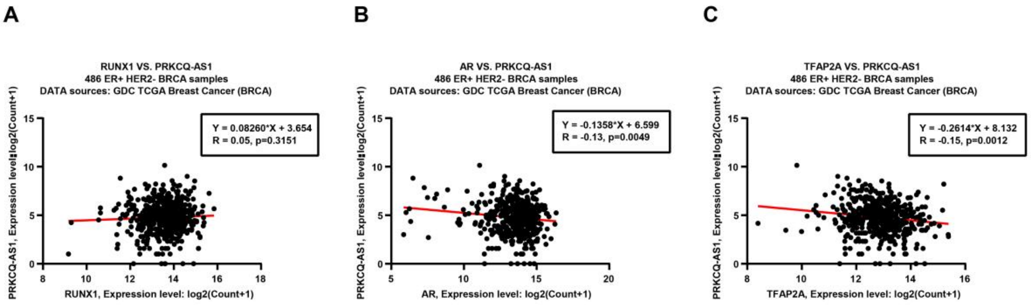

Figure S12. Correlation of expression of PKRCQ-AS1 and TFAP2A, RUNX1 or AR in breast tumor samples from GDC TCGA Breast Cancer database.

A. Correlation of expression of PKRCQ-AS1 and TFAP2A in 486 ER+ HER2- breast tumor samples from GDC TCGA Breast Cancer database.

B. Correlation of expression of PKRCQ-AS1 and RUNX1 in 486 ER+ HER2- breast tumor samples from GDC TCGA Breast Cancer database.

C. Correlation of expression of PKRCQ-AS1 and AR in 486 ER+ HER2- breast tumor samples from GDC TCGA Breast Cancer database.

GDC: genomic data commons; TCGA: the cancer genome atlas; TFAP2A: transcription factor activating enhancer binding protein 2 α; RUNX1: runt-related transcription factor 1; AR: androgen receptor; ER: estrogen receptor; HER2: human epidermal growth factor receptor 2

Figure S13

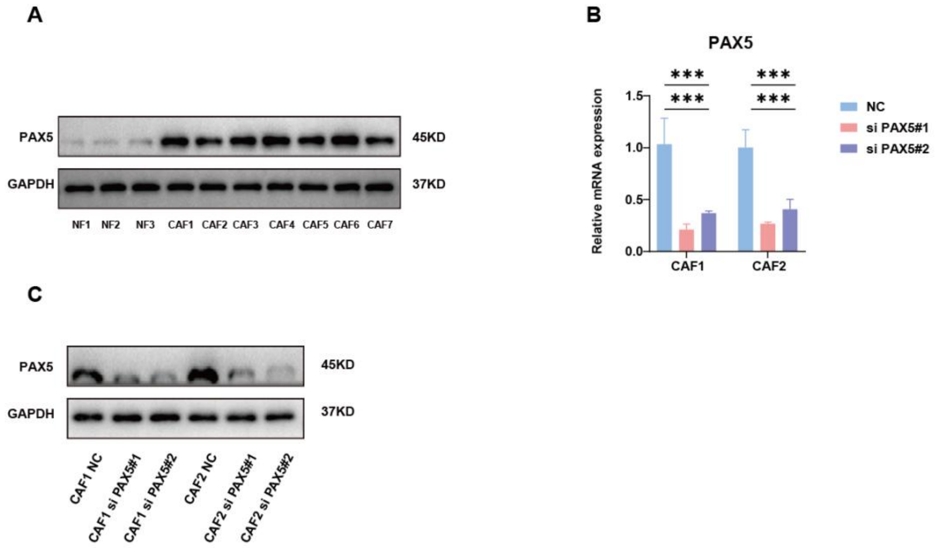

Figure S13. PAX5 downregulates in CAFs transfected with si PAX5#1 and si PAX5#2.

A. Western blots detected the expression of PAX5 in 3 NFs and 7 CAFs.

B-C. qPCR and western blots analysis of PAX5 in CAFs transfected with si PAX5#1, si PAX5#2 and negative control for 48h.

PAX5: paired box 5; NC negative control; GAPDH: glyceraldehyde-3-phosphate dehydrogenase; CAF: cancer-associated fibroblast; NF: normal fibroblast

***P<0.001. Two-tailed Student's t-test.
